# A truncated form of a transcription factor Mamo activates *vasa* in *Drosophila* embryos

**DOI:** 10.1038/s42003-019-0663-4

**Published:** 2019-11-20

**Authors:** Shoichi Nakamura, Seiji Hira, Masato Fujiwara, Nasa Miyagata, Takuma Tsuji, Akane Kondo, Hiroshi Kimura, Yuko Shinozuka, Makoto Hayashi, Satoru Kobayashi, Masanori Mukai

**Affiliations:** 1 Department of Biology, Faculty of Science and Engineering, Konan University, Okamoto, Higashinada, Kobe, 658-8501 Japan; 2 Graduate School of Natural Science, Konan University, Kobe, Japan; 3 Institute for Integrative Neurosciences, Konan University, Kobe, Japan; 4 Division of Germ Cell Biology, National Institute for Basic Biology, National Institutes of Natural Sciences, Okazaki, 444-8787 Japan; 5 Cell Biology Center, Institute of Innovative Research, Tokyo Institute of Technology, 4259 Nagatsuta-cho, Midori-ku, Yokohama, 226-8503 Japan; 6 Life Science Center for Survival Dynamics, Tsukuba Advanced Research Alliance (TARA), University of Tsukuba, Tsukuba, Ibaraki, 305-8577 Japan; 7 Graduate School of Life and Environmental Sciences, University of Tsukuba, Tsukuba, Ibaraki, 305-8572 Japan

**Keywords:** Oogenesis, Cell biology

## Abstract

Expression of the *vasa* gene is associated with germline establishment. Therefore, identification of *vasa* activator(s) should provide insights into germline development. However, the genes sufficient for *vasa* activation remain unknown. Previously, we showed that the BTB/POZ-Zn-finger protein Mamo is necessary for *vasa* expression in *Drosophila*. Here, we show that the truncated Mamo lacking the BTB/POZ domain (MamoAF) is a potent *vasa* activator. Overexpression of MamoAF was sufficient to induce *vasa* expression in both primordial germ cells and brain. Indeed, Mamo mRNA encoding a truncated Mamo isoform, which is similar to MamoAF, was predominantly expressed in primordial germ cells. The results of our genetic and biochemical studies showed that MamoAF, together with CBP, epigenetically activates *vasa* expression. Furthermore, MamoAF and the germline transcriptional activator OvoB exhibited synergy in activating *vasa* transcription. We propose that a Mamo-mediated network of epigenetic and transcriptional regulators activates *vasa* expression.

## Abstract (web summary)

Shoichi Nakamura, Seiji Hira et al. demonstrate that a truncated form of Mamo lacking the BTB/POZ domain (MamoAF) is a potent transcriptional activator of a germline gene *vasa*. This study suggests that MamoAF cooperates with transcriptional activators CBP and OvoB to regulate the development of germline cells.

## Introduction

The germline is the only cell lineage that contributes to the production of the next generation. Accordingly, germline cells exhibit properties, including stemness and longevity, that are distinct from those of somatic cells. In animals, the germline is specified by maternal determinants or inductive signals^1^. Irrespective of the mode of specification, primordial germ cells (PGCs) express conserved germline genes. *vasa* (*vas*), which encodes a DEAD-box RNA helicase^2^, is a highly conserved germline gene, and its expression is a marker of germline establishment in many species^1^. For example, Ddx4, the human ortholog of Vas, is used as a molecular biomarker for germ cells^3^. Moreover, genetic studies have demonstrated that Vas is required for gametogenesis in *Drosophila*, *C. elegans*, zebrafish and mouse^4–7^. Vas has essential roles that contribute to germline development, including translational regulation of specific mRNAs and production of Piwi-interacting RNAs^8,9^.

In *Drosophila*, maternal factors localised in the germ plasm are partitioned into PGCs to direct germline development. For example, the mitochondrial large ribosomal RNA and the Germ cell-less protein are both involved in PGC formation^10^. The maternal translational repressor Nanos is essential for repressing somatic differentiation of PGCs^11^. The Polar granule component protein has an essential role in PGC maintenance by inhibiting the positive transcription elongation factor b in newly formed PGCs^12^. The maternal Trapped in endoderm-1 protein is required for PGC migration through the midgut wall^13^. The germ plasm is necessary and sufficient for germ cell establishment^14^, suggesting that maternal transcription factors localised in the germ plasm activate *vas* expression in PGCs. Indeed, RNA interference-mediated knockdown of maternal mRNAs enriched in the germ plasm has revealed that six transcription factors are required for the expression of germline genes in PGCs^15^. Among them, the transcriptional activator OvoB, which is encoded by the *ovo* gene, is predominantly expressed in PGCs during embryogenesis, and its maternal function is essential for germline development^16^. However, the genes sufficient for *vas* activation remain unknown.

Previously, we showed that the maternal factor Mamo, which contains both a BTB/POZ domain and C_2_H_2_ Zn-finger domains and is enriched in PGCs, is necessary for *vas* expression in PGCs^17^. Mamo protein is detectable in the nuclei of PGCs at stage 15, in which *vas* is actively expressed^17^. Mamo can bind chromosomes when Mamo is expressed in salivary gland cells^18^. Biochemical analyses demonstrated that the C_2_H_2_ Zn-finger domains of Mamo directly bind specific DNA sequences^18^. C_2_H_2_ Zn-finger domains are among the most common domains in the transcription factors of metazoans^19^. The Zn-finger domains of Mamo are homologous to those of human Sp1;^20^ in vertebrates, Sp1-related transcription factors regulate gene expression in germ cells^21–24^. Accordingly, we focused on Mamo and investigated its role in *vas* activation in *Drosophila* embryos.

Biochemical analysis is an essential approach to understanding the molecular basis of transcriptional activation. To date, however, biochemical studies of *vas* activation have been difficult owing to the small number of PGCs in embryos. Accordingly, an experimental system capable of inducing forced expression of *vas* could be a powerful tool for analysing the mechanism of *vas* activation. In this study, we found that an N-terminal truncated Mamo isoform lacking the BTB/POZ domain but retaining the C_2_H_2_ Zn-finger domains was a potent *vas* activator. Maternal overexpression of the truncated Mamo (MamoAF) was sufficient to activate *vas* expression in both PGCs and brain in embryos. Indeed, Mamo mRNA encoding a truncated Mamo isoform, which is similar to MamoAF, was predominantly expressed in PGCs, in which *vas* is actively expressed. Moreover, MamoAF could partially rescue the *mamo* mutant phenotype. Therefore, through biochemical and genetic analyses in which *vas* expression was induced by MamoAF, we elucidated the molecular mechanisms of *vas* activation. MamoAF directly bound the *vas* locus and, together with CBP, epigenetically activated *vas* expression. In addition, we identified the germline transcriptional activator OvoB as a cofactor of MamoAF. MamoAF physically interacted with OvoB, and the two proteins exhibited synergy in activating *vas* transcription. Together, these findings demonstrate that Mamo-mediated transcriptional regulation directly activates *vas* expression.

## Results

### Mamo short isoform may be associated with *vas* activation

**Fig. 1:**
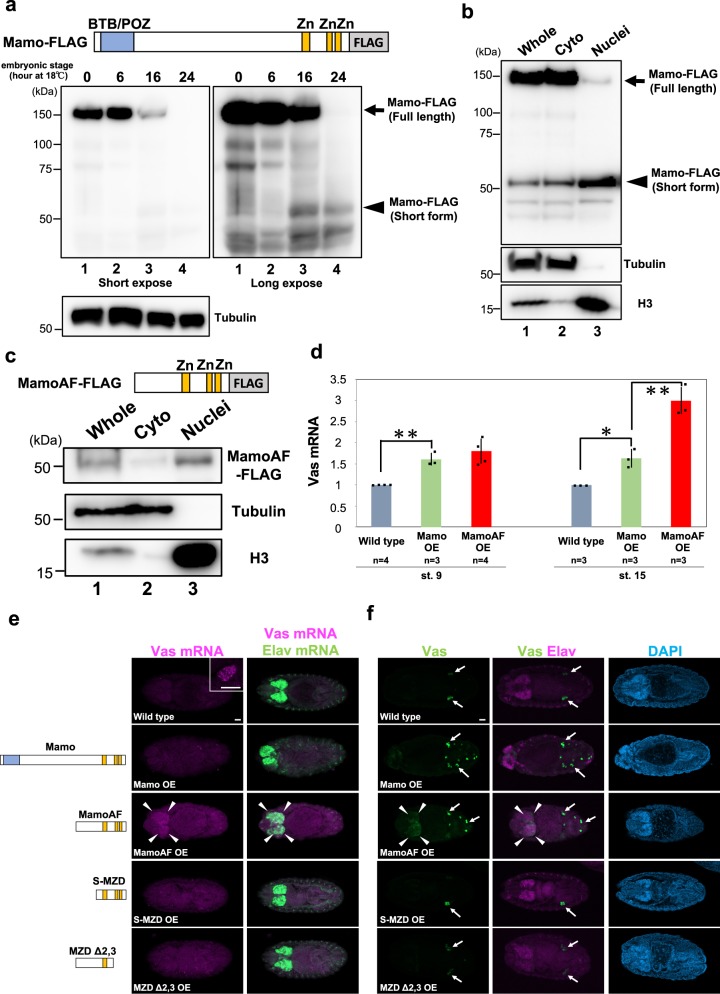
MamoAF overexpression induces *vas* expression in embryos. **a** Developmental western blotting analysis of embryos expressing full-length Mamo-FLAG under the control of *matα4-Gal-VP16* driver. Embryos were cultured at 18 °C for indicated hours and subjected to western blotting with anti-FLAG antibody. Embryos cultured at 18 °C for 0, 6, 16 and 24 h are corresponded to embryonic stage 4, 9, 12 and 15, respectively. **b** Analysis of fractionated samples extracted from embryos expressing full-length Mamo-FLAG by western blotting. Embryos expressing Mamo-FLAG under the control of *matα4-Gal-VP16* driver were cultured at 25 °C for 0–16 h and fractionated. Normalised load of each extract (7 μg) were analysed by western blotting with anti-FLAG, anti-Tubulin and anti-Histone H3 antibodies. Whole extract (lane 1), cytoplasmic extract (lane 2) and nuclear extract (lane 3) were loaded. **c** Analysis of fractionated samples extracted from embryos expressing MamoAF-FLAG by western blotting. Embryos expressing MamoAF-FLAG under the control of *mat4α-Gal-VP16* driver were cultured at 25 °C for 0–16 h and fractionated. Normalised load of each extract (11 μg) were analysed by western blotting with anti-FLAG, anti-Tubulin and anti-Histone H3 antibodies. Whole extract (lane 1), cytoplasmic extract (lane 2) and nuclear extract (lane 3) were loaded. Full images of the electrophoreses are shown in Supplementary Fig. 16. **d** qRT-PCR analyses of Vas mRNA in wild type, Mamo and MamoAF overexpressed embryos under the control of *matα4-Gal-VP16* driver. Error bars are SD of the mean. **P < 0.01, *P < 0.05 (two-tailed Student’s *t* test). **e** Double fluorescence in situ hybridisation for Vas and Elav mRNAs of wild type, the embryos overexpressing Mamo, MamoAF and MamoAF fragments under the control of *matα4-Gal-VP16* driver. Elav is a neural marker. Confocal sections of the brain of the embryos are shown. The inset shows close-up image of wild-type PGCs. **f** Double-staining for Vas and neural marker Elav of wild type, the embryos overexpressing Mamo, MamoAF and MamoAF fragments under the control of *matα4-Gal-VP16* driver. Arrows indicate PGCs expressing Vas. Arrowheads indicate ectopic Vas expression. Scale bar: 30 μm

To evaluate the molecular function of Mamo protein, we expressed a C-terminally FLAG-tagged allele of Mamo that can rescue the *mamo* mutant phenotype^17^ in embryos, under the control of the maternal Gal4 driver (*matα4-Gal-VP16*). During embryogenesis, maternally expressed FLAG-tagged Mamo (~ 150 kDa) was processed into shorter proteins (~ 50 kDa) that lacked the N-terminal BTB/POZ domain but retained the C-terminal Zn-finger DNA-binding domains (Fig. 1a). Full-length Mamo, as well as its ~ 100-kDa and ~ 75-kDa derivatives, was detected in early embryos, in which zygotic *vas* expression is not yet activated (Fig. 1a, lanes 1 and 2). By contrast, in later embryos, in which zygotic *vas* expression is activated, the level of full-length Mamo was considerably reduced, and the ~ 50-kDa derivatives of Mamo became detectable (Fig. 1a, lanes 3 and 4). These observations imply that maternally provided Mamo is gradually processed into shorter derivatives through specific proteolytic processes. Moreover, in contrast to full-length Mamo, short derivatives of Mamo preferentially accumulated in the nuclear fraction (Fig. 1b). Based on these observations, we hypothesised that the short isoform of Mamo, which lacks the BTB/POZ domain but retains the Zn-finger domains, (hereafter, referred to as Mamo short isoform) is a potent *vas* activator.

### Mamo mRNA encoding Mamo short isoform is expressed in PGCs

Next, we investigated whether Mamo mRNA encoding the Mamo short isoform is expressed in PGCs. Expressed sequence tags (GM29022 and GM29051) have shown that the short Mamo mRNA is expressed in ovaries^20^. 5′-rapid amplification of cDNA ends (RACE) confirmed that short Mamo mRNA is expressed in ovaries. The sequences of 5′-RACE clones revealed that the short Mamo mRNA encodes the Mamo short isoform, which lacks the BTB/POZ domain, and arises owing to the use of alternative transcription initiation sites (Supplementary Fig. 1). To investigate the expression patterns of short Mamo mRNA, we performed in situ hybridisation using a Mamo Zn-finger probe, which detects both short and full-length Mamo mRNAs, and a Mamo BTB probe, which specifically detects the full-length Mamo mRNA (Supplementary Fig. 2a). Mamo mRNA signals were detected in germ cells in the ovaries when using a Mamo Zn-finger probe or Mamo BTB probe (Supplementary Fig. 2b), confirming that these probes are available. These results are consistent with previous studies showing that full-length Mamo mRNA is expressed in the ovary^17,20^. By contrast, during embryogenesis, Mamo mRNA signals were specifically detected in PGCs only when using a Mamo Zn-finger probe, but not a Mamo BTB probe (Supplementary Fig. 2b). In the early embryos, at stages 5–9, no Mamo mRNA signal was detected, whereas at stages 14–16, the Mamo mRNA signals were more intense in PGCs, suggesting that Mamo mRNA encoding the Mamo short isoform lacking the BTB/POZ domain is zygotically expressed in PGCs, in which *vas* is actively expressed. Antibodies specific for the Mamo short isoform are not yet available, because the amino-acid sequence of the Mamo short isoform is present within that of full-length Mamo. These observations indicate that Mamo short isoform is predominantly expressed in PGCs, and that this isoform may promote *vas* expression.

### MamoAF can partially rescue the *mamo* mutant phenotype

Because a *mamo* mutant specific for the short isoform was not available, we used overexpression experiments to evaluate the function of the short isoform. Previously, we generated a UAS line (UAS-MZD-FLAG) to produce a truncated Mamo protein lacking the BTB/POZ domain^18^ (hereafter, referred to as MamoAF). When it was expressed in embryos under the control of *matα4-Gal-VP16*, MamoAF preferentially accumulated in the nuclear fraction (Fig. 1c), just like the short derivatives of Mamo (Fig. 1b). Next, we investigated whether MamoAF overexpression can rescue the *mamo* mutant phenotype. Egg chambers containing the germ cells derived from *mamo* mutant germline clones were rarely observed in ovarioles (1.4%, *n* = 74) (Supplementary Fig. 3, Supplementary Table 1). However, when full-length Mamo-FLAG or MamoAF-FLAG was expressed in germ cells under the control of the *nanos-Gal4-VP16 (nos-Gal4)* driver, differentiating egg chambers containing germ cells derived from *mamo* mutant germline clones after oogenic stage 6 were observed in 28.8% and 13.2% of ovarioles, respectively (Supplementary Fig. 3, Supplementary Table 1). Mamo or MamoAF overexpression could rescue the *mamo* mutant phenotype (*P* < 0.01, Fisher’s exact test). We speculated that the low rescue activity of Mamo may be due to the expression level of Mamo driven by *nos-Gal4*, which is different from the endogenous *mamo* promoter. Next, we investigated whether MamoAF overexpression can rescue the differentiation of *mamo* mutant germ cells into mature eggs. Overexpression of full-length Mamo, but not MamoAF, rescued the differentiation of *mamo* mutant germ cells into mature eggs (Supplementary Table 2). To assess the difference in rescue ability between full-length Mamo and MamoAF, we examined the expression patterns of full-length Mamo-FLAG or MamoAF-FLAG in ovaries when these proteins were expressed under the control of *nos-Gal4*. Full-length Mamo was enriched both in the nuclei of germline cysts and in the nuclei of the nurse cells in egg chambers after oogenic stage 6 (Supplementary Fig. 4). In 65.3% of egg chambers at stage 6, full-length Mamo signals were enriched in the nuclei of the nurse cells (*n* = 121): 84.3% at stage 7 (*n* = 115). By contrast, MamoAF was detected in the nuclei of germline cysts, whereas MamoAF signals were weak in the nuclei of nurse cells in egg chambers (Supplementary Fig. 4). In 25.3% of egg chambers at stage 6, MamoAF signals were enriched in the nuclei of the nurse cells (*n* = 87): 18.1% at stage 7 (*n* = 72). Together, these findings show that MamoAF could partially rescue the differentiation of *mamo* mutant germline clones. Accordingly, we used overexpression of MamoAF to investigate the effect of the Mamo short isoform on *vas* activation.

### MamoAF is sufficient to activate *vas* expression

To determine whether MamoAF can induce *vas* expression, we monitored *vas* expression in embryos overexpressing full-length Mamo or MamoAF under the control of *matα4-Gal-VP16* driver. Quantitative reverse-transcription PCR (qRT-PCR) revealed that maternal overexpression of full-length Mamo only moderately increased *vas* expression at stages 9 and 15, whereas MamoAF markedly increased expression of *vas* at stage 15 (Fig. 1d). In addition, we observed somatic *vas* expression, especially in brain of embryos overexpressing MamoAF (hereafter, referred to as MamoAF-OE embryos), but not in embryos overexpressing full-length Mamo (Fig. 1e, f). In MamoAF-OE embryos, PGCs formed normally at the posterior poles, and brain cells expressing Vas appeared at stage 14 (Supplementary Fig. 5). These results show that MamoAF overexpression is sufficient to induce *vas* expression in brain.

Next, we investigated whether MamoAF can promote *vas* expression in PGCs. Specifically, we monitored *vas* expression in PGCs when MamoAF was overexpressed under the control of the maternal *nos-Gal4* driver^16^. MamoAF overexpression increased *vas* expression in PGCs at stages 14 and 15 (Supplementary Fig. 6). We also found that overexpression of full-length Mamo increased *vas* expression in PGCs at stage 15 (Supplementary Fig. 6). These results indicate that the Mamo short isoform is a potent inducer of *vas* expression in PGCs.

### The *mamo* gene produces two types of *vas* activators

Full-length Mamo could promote *vas* expression in PGCs, but its activity was weaker than that MamoAF (Supplementary Fig. 6). In embryos, full-length Mamo preferentially accumulated in the cytoplasmic fraction relative to MamoAF (Fig. 1b), which may suppress its transcriptional activity. Thus, the BTB/POZ domain may regulate the nuclear localisation of full-length Mamo. Consistent with this notion, overexpression of full-length Mamo did not strongly induce *vas* expression in brain (Fig. 1e, f). These results suggest that full-length Mamo is a weak but specific activator of *vas* whose activity is restricted in PGCs. By contrast, MamoAF lacking the BTB/POZ domain preferentially accumulated in the nuclear fraction (Fig. 1c), and potently induced *vas* in PGCs and brain. Vas mRNA becomes detectable in PGCs at stage 9, when short Mamo mRNAs are not yet zygotically expressed in PGCs. Thus, we propose that maternal full-length Mamo establishes specific *vas* expression in early PGCs at stage 9. In later PGCs, after stage 14, zygotic Mamo short isoform contributes to the maintenance of robust *vas* expression. We found that, during embryogenesis, maternally expressed full-length Mamo was processed into shorter proteins (Fig. 1a). Thus, in addition to zygotic Mamo short isoform, the conversion from maternal full-length Mamo into short derivatives may promote *vas* expression in late PGCs.

### Zn-finger domain of MamoAF is required for *vas* activation

To investigate the mechanisms underlying *vas* activation, we focused subsequent experiments on MamoAF owing to its potency as a *vas* activator. To evaluate the functional domains of MamoAF, we examined *vas* expression in embryos expressing fragments of the protein. Fragments of MamoAF proteins that lacked the N-terminal region (S-MZD) or the C-terminal Zn-finger DNA-binding domains (MZDΔ2, 3) failed to induce *vas* expression in brain (Fig. 1e, f), indicating that both of these domains are required to induce somatic *vas* expression. Thus, the Zn-finger domains of MamoAF may directly bind to *vas*.

### MamoAF directly binds and epigenetically activates *vas*

To determine whether MamoAF directly binds *vas*, we performed electrophoretic mobility shift assays (EMSA) and chromatin immunoprecipitation assays (ChIP) on MamoAF-OE embryos. Although the upstream region (~ 2 kbp) of the *vas* gene does not contain cis-elements containing a Mamo-binding consensus sequence^18^, the first intron of *vas* contains several possible cis-elements, suggesting that MamoAF activates *vas* through previously unidentified cis-elements in the first intron. One such cis-element, hereafter referred to as the *vas*-A element, is conserved among several *Drosophila* species (Supplementary Fig. 7). Hence, we focused subsequent experiments on the *vas*-A element.

**Fig. 2:**
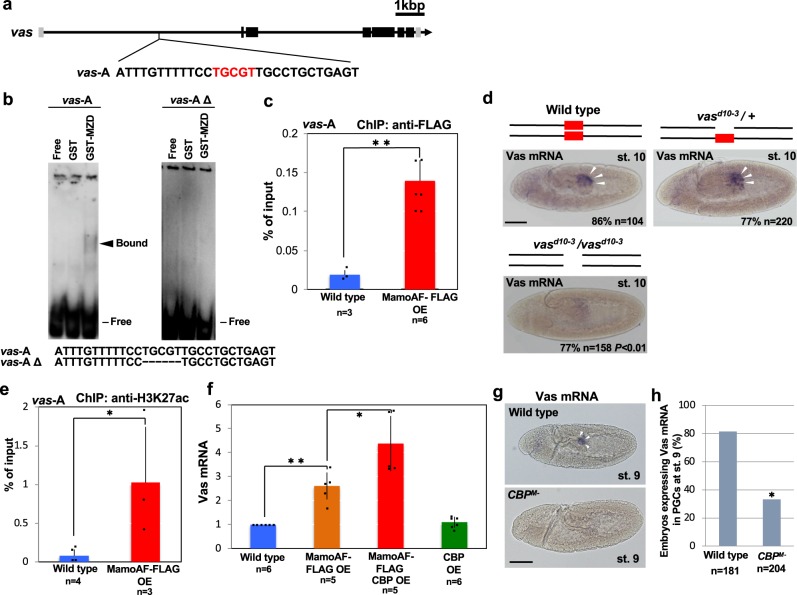
MamoAF directly binds *vas* and epigenetically activates *vas* together with CBP. **a** *vas*-A element, which contains a Mamo-binding consensus sequence (red), in the first intron of *vas*. **b** EMSA analyses of Zn-finger domains of Mamo protein (MZD) using *vas*-A and *vas*-AΔ probes. *vas*-AΔ probe lacks a Mamo-binding consensus sequence. **c** ChIP-qPCR assays were performed with MamoAF-OE embryos and FLAG antibody. The precipitated DNA was detected by qPCR with primers targeting to *vas-*A. Error bars are SD of the mean. **P < 0.01 (two-tailed Student’s *t* test). **d** Vas mRNA in situ hybridisation of wild type, *vas*^*d10-3*^ / + and *vas*^*d10-3*^ / *vas*^*d10-3*^ embryos at stage 10. The Mamo-binding consensus sequence (red box) is deleted from *vas*-A element in *vas*^*d10-3*^ / *vas*^*d10-3*^embryo. Scale bar: 50 μm. **e** ChIP-qPCR assays were performed with MamoAF-OE embryos and H3K27ac antibody. The precipitated DNA was detected by qPCR with primers targeting to *vas-*A. Error bars are SD of the mean. *P < 0.05 (one-tailed Student’s *t* test). **f** qRT-PCR analyses of Vas mRNA in wild type, MamoAF, CBP and both CBP and MamoAF overexpressed embryos. Error bars are SD of the mean. *P < 0.05, **P < 0.01 (two-tailed Student’s *t* test). **g** Vas mRNA in situ hybridisation of wild type and *CBP*^*M*-^ embryo derived from *nej*^*0.3*^ mutant germline clones at stage 9. White arrowheads indicate PGCs expressing Vas mRNA. Scale bar: 50 μm. **h** Percentages of embryos expressing Vas mRNA in wild type and *CBP*^*M*-^ embryos at stage 9. *P < 0.01 (Fisher’s exact test)

EMSA revealed that the Zn-finger domains of MamoAF directly bound the *vas*-A element in the first intron of *vas* (Fig. 2a, b), and ChIP-qPCR assays confirmed that MamoAF bound to this sequence in vivo (Fig. 2c). Next, we investigated whether the *vas*-A element is required for *vas* expression. After removal of the Mamo-binding sequence by gene editing, *vas* expression in PGCs was considerably reduced (Fig. 2d; Supplementary Fig. 8). Vas mRNA signals were detected in PGCs of 86% of wild-type embryos (*n* = 104) and 77% of embryos heterozygous for *vas*^*d10-3*^ (*n* = 220), but only 23% of embryos homozygous for *vas*^*d10-3*^, which lacks the *vas*-A element (*n* = 158, *P* < 0.01). Together, these results demonstrate that MamoAF directly activates *vas* through the *vas-*A element.

### MamoAF works with CBP to epigenetically activate *vas*

We hypothesised that the *vas*-A element acts as an enhancer. If so, acetylated H3K27 (H3K27ac), which marks active enhancers^25–27^, should accumulate in this region. ChIP assays revealed greater enrichment of H3K27ac at the *vas*-A element in MamoAF-OE than in wild-type embryos, suggesting that MamoAF activates *vas-*A through H3K27ac (Fig. 2e). Because MamoAF lacks a histone acetyltransferase (HAT) domain, MamoAF must collaborate with CBP, which is responsible for acetylation of H3K27 in *Drosophila*^25^. Accordingly, we investigated the genetic interaction between MamoAF and CBP. Because CBP is essential for embryogenesis, we examined the outcome of CBP overexpression. Overexpressing both CBP and MamoAF synergistically increased *vas* expression, whereas CBP overexpression alone did not (Fig. 2f). These results demonstrate that MamoAF collaborates with CBP to epigenetically activate *vas*.

Next, we investigated whether CBP is required for *vas* expression in PGCs. In *Drosophila*, CBP is encoded by *nejire* (*nej*)^25^. In embryos derived from *nej* germline clones, maternal CBP activity was required for both H3K27ac accumulation (Supplementary Fig. 9) and *vas* expression in PGCs (Fig. 2g, h). These results show that *vas* expression in PGCs is epigenetically activated through H3K27ac. These findings are consistent with our idea that Mamo collaborates with CBP to epigenetically activate *vas* in PGCs.

### MamoAF collaborates with OvoB

**Fig. 3:**
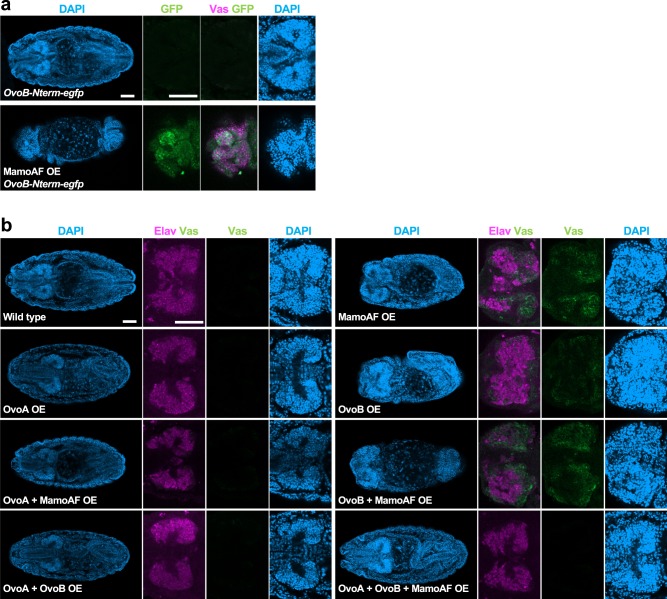
MamoAF collaborates with OvoB to induced Vas expression in brain. **a** Immunostaining of embryos carrying *OvoB-Nterm-egfp* allele and MamoAF OE carrying *OvoB-Nterm-egfp* allele showing MamoAF induces ectopic GFP expression from the *OvoB-Nterm-egfp* allele. MamoAF is overexpressed under the control of maternal *matα4-Gal-VP16* driver. Scale bar: 50 μm. **b** Wild type, MamoAF OE, OvoA OE, OvoB OE, OvoA + MamoAF OE, OvoB + MamoAF OE, OvoA + OvoB OE and OvoA + OvoB + MamoAF OE are stained for Vas, Elav and DAPI. The transgenes are overexpressed under the control of maternal *matα4-Gal-VP16* driver. Whole embryos are shown in left, close-up views of brain of the embryos are in right panels. Scale bar: 50 μm

In addition to CBP, we assumed that a transcriptional activator collaborates with MamoAF for *vas* activation, because MamoAF lacks a transcriptional activation domain. The transcriptional activator OvoB, which is maternally expressed and maintained predominantly in PGCs, is required for *vas* expression^15,16^, making it a candidate cofactor of Mamo. However, because OvoB activity is essential for oogenesis^28^, loss-of-function alleles of *ovo* are not available for investigating the role of maternal OvoB in *vas* activation. To overcome this problem, we used MamoAF-induced *vas* expression to determine whether OvoB cooperates with MamoAF to promote *vas* expression. If OvoB is essential for *vas* expression, then MamoAF should induce OvoB expression in brain. We examined the effects of MamoAF on OvoB expression using the *ovoB-Nterm-egfp* knock-in allele, which enabled us to detect OvoB as GFP fluorescence^16^ (Supplementary Fig. 10a). As expected, MamoAF overexpression induced GFP expression from the *ovoB-Nterm-* *egfp* allele, especially in brain expressing *vas* (Fig. 3a). The *ovo* locus produces three proteins: OvoA and OvoB, which serve, respectively, as a transcriptional repressor and activator in germ cells, and Shavenbaby (Svb), which regulates epidermal differentiation^29^. In situ hybridisation of MamoAF-OE embryos detected Ovo mRNA signals in brain when using an Ovo common probe, but not an Svb-specific probe (Supplementary Fig. 10b–q). Because OvoA is a transcriptional repressor, MamoAF probably induces OvoB in brain expressing *vas*. Although the mechanism remains elusive, these observations show that MamoAF induces OvoB expression in brain. Therefore, MamoAF may collaborate with OvoB transcriptional activator to promote *vas* expression in brain.

The OvoA transcriptional repressor can antagonise OvoB-induced transcription;^16,28^ therefore, if MamoAF collaborates with OvoB, then OvoA should suppress MamoAF-induced *vas* expression. Indeed, OvoA overexpression suppressed MamoAF-induced *vas* expression in brain. No somatic Vas expression was observed in embryos overexpressing both MamoAF and OvoA (*n* = 147) (Fig. 3b). We also confirmed the absence of somatic Vas expression in wild-type embryos (*n* = 175), embryos expressing OvoA (*n* = 116), embryos expressing both OvoA and OvoB (*n* = 82), and embryos expressing OvoA, OvoB and MamoAF (*n* = 35) (Fig. 3b). Taken together, these results show that MamoAF collaborates with OvoB to induce Vas expression in brain.

Because MamoAF induces OvoB expression in brain (Fig. 3a, Supplementary Fig. 10b–q), OvoB may act downstream of MamoAF. Thus, we tested whether OvoB is sufficient to induce Vas expression in brain. We evaluated *vas* expression in embryos overexpressing OvoB. Strong Vas signals in brain were detected in MamoAF-OE embryos (58.3%, *n* = 127), but not in OvoB-OE embryos (*n* = 71). OvoB overexpression induced much weaker somatic Vas expression (35.2%, *n* = 71) than MamoAF-OE embryos (Fig. 3b). This observation indicates that OvoB alone is not sufficient to induce strong Vas expression in brain. Vas expression in brain was increased in embryos expressing both MamoAF and OvoB compared with embryos expressing OvoB alone. The increased Vas expression in brain appears to be owing to the enhancement of OvoB expression induced by MamoAF. However, Vas expression in brain of embryos expressing both MamoAF and OvoB (66.7%, *n* = 63) was not significantly increased relative to embryos expressing MamoAF alone (58.3%, *n* = 127) (Fig. 3b). This indicates that the promotion of *vas* expression in brain cannot be explained by the increased expression of OvoB alone. These results suggest that MamoAF collaborates with OvoB to promote *vas* expression in brain.

**Fig. 4:**
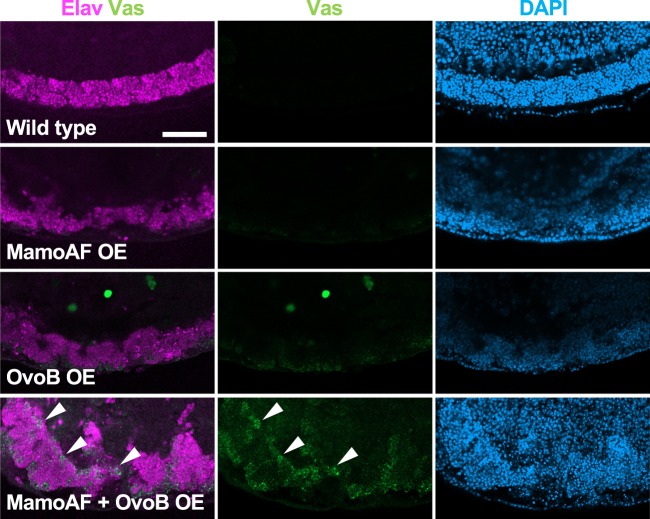
Overexpression of both MamoAF and OvoB is sufficient to induce Vas expression in VNC. Wild type, MamoAF OE, OvoB OE and MamoAF + OvoB OE are stained for Vas, Elav and DAPI. The transgenes are overexpressed under the control of maternal *matα4-Gal-VP16* driver. Close-up views of ventral region of embryos are shown. White arrowheads indicate ectopic Vas expression in the VNC. Scale bar: 50 μm

Given that the collaboration between MamoAF and OvoB are essential for *vas* activation, overexpression of both MamoAF and OvoB should activate *vas* expression in somatic cells, where MamoAF cannot induce OvoB. Indeed, overexpression of both MamoAF and OvoB synergistically induced Vas expression in somatic cells in the ventral nerve cord (VNC) (55.6%, *n* = 63), a region where no expression was observed in embryos expressing MamoAF or OvoB alone (*n* = 127, *n* = 71, respectively) (Fig. 4). Overexpression of both MamoAF and OvoB is sufficient for activating *vas* expression in VNC. However, other somatic cells exhibited little response to MamoAF and OvoB. Thus, *vas* expression in somatic cells is dependent on their cellular contexts. Although the pertinent differences in cellular context remain elusive, these observations show that both MamoAF and OvoB are essential for *vas* activation both in brain and VNC.

**Fig. 5:**
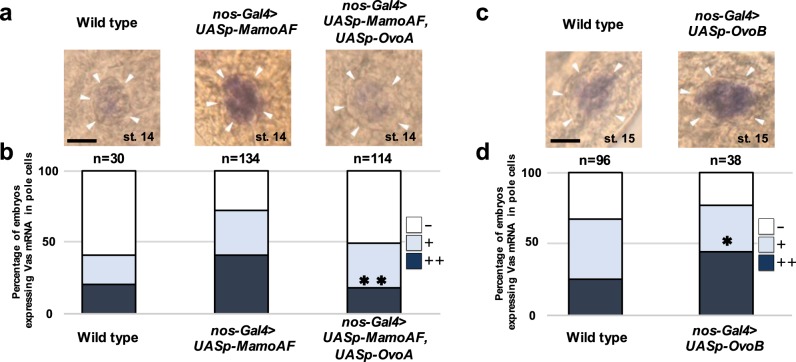
MamoAF collaborates with OvoB to induce Vas expression in PGCs. **a** Vas mRNA in situ hybridisation of wild type, MamoAF overexpressed and MamoAF and OvoA overexpressed embryos under the control of maternal *nos-Gal4* driver at stage 14. White arrowheads indicate embryonic gonads. **b** Embryos were classified into three groups depending on their strong (++), middle (+) and low (−) signal intensities of Vas mRNA in PGCs. Percentages of the embryos carrying PGCs with strong, middle and low signals in wild type, MamoAF-OE and MamoAF + OvoA OE embryos. **P < 0.01 (Significance is calculated with MamoAF OE by Fisher’s exact test). **c** Vas mRNA in situ hybridisation of wild type and OvoB overexpressed embryos under the control of maternal *nos-Gal4* driver at stage 15. White arrowheads indicate embryonic gonads. **d** Embryos were classified into three groups depending on their strong (++), middle (+) and low (−) signal intensities of Vas mRNA in PGCs. Percentages of the embryos carrying PGCs with strong, middle and low signals in wild type and OvoB-OE embryos. *P < 0.05 (Significance is calculated with wild type by Fisher’s exact test). Scale bar: 20 μm

Next, we investigated whether Mamo collaborates with OvoB to promote *vas* expression in PGCs. Because OvoB activity is essential for oogenesis^28^, genetic approaches using loss-of-function alleles of *ovo* are not available for investigating the role of maternal OvoB in PGCs^16^ of progeny. To circumvent this obstacle, we paternally introduced the *UASp-ovoA* transgene into embryos derived from female adults carrying both *UASp-MamoAF* and the *nos-Gal4* driver. We found that OvoA suppressed MamoAF-induced *vas* expression in PGCs (Fig. 5a, b), and that OvoB overexpression was sufficient to promote *vas* expression (Fig. 5c, d). These results show that Mamo collaborates with OvoB to promote *vas* in PGCs.

**Fig. 6:**
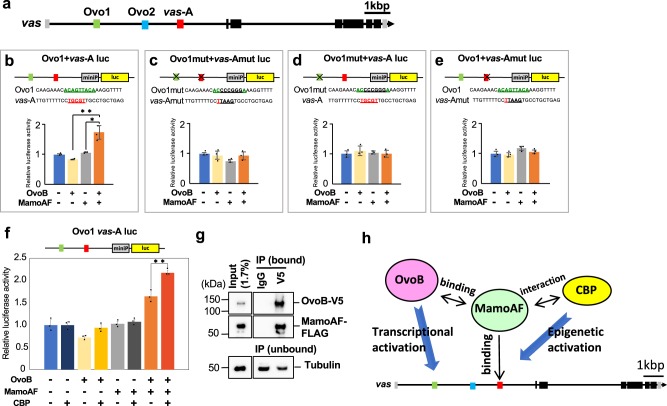
MamoAF and OvoB exhibit transcriptional synergy. **a** *vas*-A element and the Ovo-binding consensus sequences (Ovo1 and Ovo2) in the first intron of *vas*. **b**–**e** Luciferase reporter assays in S2 cells, showing that both Ovo1 and *vas*-A element are required for transcriptional activation of luciferase. All experiments were conducted as triplicate and the mean of relative luciferase activities and SD are shown. *P < 0.05, **P < 0.01 (two-tailed Student’s *t* test). **f** Luciferase reporter assays in S2 cells, showing that MamoAF, OvoB and CBP collectively stimulates luciferase expression. **P *<* 0.01 (two-tailed Student’s *t* test). All experiments were conducted as triplicate and the mean of relative luciferase activities and SD are shown. **g** Coimmunoprecipitation assays with nuclear extracts of S2 cells that had been transfected with both FLAG-tagged MamoAF and V5-tagged OvoB. The precipitate with a V5 antibody contains not only OvoB but also MamoAF-FLAG, showing MamoAF physically interacts with OvoB. Full images of the electrophoreses are shown in Supplementary Fig. 16. **h** A model of interaction between MamoAF, CBP and OvoB in the activation of *vas* expression

If MamoAF collaborates with OvoB to activate *vas*, Ovo enhancers^30^ should promote *vas* expression. In addition to the *vas*-A element required for Mamo binding (Fig. 2b), the first intron of *vas* contains Ovo-binding consensus sequences (Ovo1 and Ovo2)^30^ (Fig. 6a), which are conserved in several *Drosophila* species (Supplementary Fig. 7). To investigate whether these cis-elements have enhancer activity, we performed luciferase reporter assays in *Drosophila* Schneider cells (S2 cells). We evaluated the effects of fragments of the first intron containing each cis-element (Ovo1, Ovo2 and *vas*-A) on luciferase expression, and found that none of the fragments were sufficient to promote luciferase expression in response to MamoAF and OvoB (Supplementary Figs 11, 12). These results suggested that, individually, none of the cis-elements has enhancer activity. Next, we examined combinations of these fragments containing the cis-elements. Although a reporter construct containing both Ovo2 and *vas-*A (Ovo2 + *vas-*A luc) did not exhibit enhancer activity (Supplementary Fig. 12d), a reporter construct containing both Ovo1 and *vas-*A (Ovo1 + *vas-*A luc) promoted luciferase expression in response to both MamoAF and OvoB (Fig. 6b). Furthermore, mutation analyses of Ovo1 and *vas-*A demonstrated that both Ovo1 and the *vas-*A element were required to stimulate transcription (Fig. 6c–e). Consistent with this, both MamoAF and OvoB were required to promote luciferase expression mediated by Ovo1 and *vas*-A (Fig. 6b). We concluded that MamoAF collaborates with OvoB to activate *vas* through Ovo1 and the *vas*-A element. Furthermore, CBP enhanced luciferase expression mediated by MamoAF and OvoB, suggesting that these factors worked together to stimulate transcription (Fig. 6f).

### MamoAF physically interacts with OvoB

Next, we investigated the physical interaction between MamoAF and OvoB. Coimmunoprecipitation assays with nuclear extracts of S2 cells transfected with both FLAG-tagged MamoAF and V5-tagged OvoB revealed a physical interaction between MamoAF and OvoB (Fig. 6g; Supplementary Figs 13–15). We found that the N-terminal region encompassing residues 251–277 of MamoAF is necessary for the physical interaction with OvoB (Supplementary Fig. 14). Conversely, the region encompassing residues 470–757 of OvoB is required for the interaction with MamoAF (Supplementary Fig. 15). These results, together with the data from the luciferase reporter assay, demonstrate that MamoAF and OvoB exhibit transcriptional synergy in activating *vas* expression.

## Discussion

Although it is well recognised that maternal translational and transcriptional repressors play essential roles in establishing PGCs in *Drosophila*^11,12^, the genes sufficient for *vas* activation in PGCs remain unknown. In this study, we identified two types of *vas* activators: full-length Mamo, a weak but specific inducer of *vas*, and Mamo short isoform, a potent inducer of *vas* activation. To clarify the molecular mechanisms of *vas* activation, we conducted biochemical and genetic analyses using MamoAF-induced *vas* expression, and revealed that two cofactors of MamoAF, CBP and OvoB, are both involved in activation of *vas* in PGCs. Thus, MamoAF-induced *vas* expression is useful for identifying cofactors of *vas* activation in PGCs. MamoAF can induce *vas* expression in both PGCs and brain. In both cellular contexts, the transcriptional activator OvoB is necessary for MamoAF-induced *vas* expression. Moreover, overexpression of both MamoAF and OvoB is sufficient to induce *vas* expression in VNC. Thus, the Mamo–OvoB axis is essential for directing *vas* activation. Our data revealed that MamoAF functions as a molecular hub: it collaborates with CBP to epigenetically activate the *vas* locus, and physically interacts with OvoB to stimulate *vas* transcription (Fig. 6h). Consistent with this notion, a reporter assay demonstrated that these factors worked together to stimulate transcription. We conclude that the Mamo-mediated network of epigenetic and transcriptional regulators directs *vas* activation in *Drosophila* embryos.

We successfully demonstrated that MamoAF directly activates *vas* expression through the *vas-*A element in the first intron, which is essential for endogenous *vas* expression in PGCs. However, MamoAF could also activate *vas* transcription through other cis-elements that remain to be identified. We previously reported that Mamo has a role in the regulation of chromatin structure^18^. Therefore, Mamo may regulate chromatin structure, in addition to transcriptional activation, to promote *vas* expression.

A previous study using reporter assays showed that a 40-bp element in the 5′ flanking region of *vas*, the *up-40* element, is sufficient to recapitulate germline-specific expression during oogenesis and embryogenesis^31^. The *up-40* element does not contain consensus sequences for either Mamo^18^ or Ovo^30^. This implies that different transcriptional factors must control *vas* expression through the *up-40* element. Because removal of the *vas-*A element decreases endogenous *vas* expression in PGCs, *vas-*A and *up-40* elements may act in a partially redundant manner to upregulate *vas* expression in PGCs. Therefore, multiple enhancers may act in parallel to activate *vas* expression in germ cells. However, it remains unclear whether the *up-40* element is necessary for endogenous *vas* expression.

In this study, we focused on MamoAF to investigate the mechanisms of *vas* activation due to its potent activity for *vas* activation. Thus, it remains unclear how full-length Mamo activates *vas* expression. Understanding the mechanism requires the identification of factors regulating the nuclear localisation of full-length Mamo. Maternal full-length Mamo in the nuclei of early PGCs may interact with OvoB, which is maternally provided and enriched in PGC nuclei^16^. However, it will be necessary to investigate the interaction between full-length Mamo and OvoB under conditions in which full-length Mamo is not converted into short derivatives.

Both full-length and short form Mamo mRNAs are expressed in germ cells in ovaries. However, full-length Mamo rescues the differentiation of *mamo* mutant germline clones more efficiently than MamoAF. We found that full-length Mamo can be converted to truncated derivatives. Thus, full-length Mamo has the potential to complement the short isoform. By contrast, the short form of Mamo does not appear to complement the function of the full-length protein owing to the lack of the BTB/POZ domain. During oogenesis, the transcriptional activity of full-length Mamo may be regulated through interactions with epigenetic regulators via the BTB/POZ domain, as reported for other BTB/Zn-finger transcription factors^32^. Full-length Mamo is enriched in the nuclei of the nurse cells in egg chambers after oogenic stage 6, but MamoAF is only weakly enriched. Thus, full-length Mamo may play a role in regulating gene expression in nurse cells and promoting the differentiation of oocytes into mature eggs.

We propose that Mamo short isoform is a potent *vas* activator. The C_2_H_2_ Zn-finger domains of Mamo short isoform are homologous to those of human Sp1;^20^ Sp1-related transcription factors regulate gene expression in germ cells in vertebrates^21–23^. For example, chicken Sp1 promotes *vas* expression in PGCs^24^. Moreover, Ovo has conserved roles in germline development in mouse and *Drosophila*^16,33^. Accordingly, Sp1-related Zn-finger proteins and Ovo may also be key transcriptional activators of *vas* in other animals, including mouse and human. We anticipate that our results will facilitate understanding of the molecular mechanisms that regulate germline development in animals.

## Methods

### Fly stains

*Oregon-R* served as a wild-type control. *UASp-Mamo-FLAG* has been described previously^17^. *UASp-MZD-FLAG*^18^ was used to overexpress MamoAF. *nos-Gal4* was a gift from Dr. R. Lehmann. *UAS-CBP* was a gift from Dr. J. Kumar. *w ovo*^*D1*^ *v*^24^ *P{FRT}101;P{hsFLP}38* (BL1813), *matα4-Gal-VP16* (BL7063)*, nej*^*0.3*^(BL34040)*, nej*^*3*^(BL3729) were obtained from the Bloomington Stock Center. *ovoB-Nterm-egfp, UASp-OvoB* and *UASp-OvoA* has been described previously^16^. All stocks were maintained at 25 °C or at room temperature in standard *Drosophila* medium unless otherwise noted.

### Expression vectors for Mamo fragments in embryos

The fragment of s-MZD-FLAG was amplified from the pBluescript-*mamo*-FLAG plasmid^17^ by PCR. The fragment of MZDΔ2,3-FLAG was amplified from the pUASp-MZD-FLAG plasmid^18^ by PCR. For s-MZD-FLAG, p-UASp-d2,3 F (5′-CGCGGATCCATG CCACTGCACATGTTTCCG-3′) and T3 (5′-AATTAACCCTCACTAAAGGG-3′) primers were used. For MZDΔ2,3-FLAG, UASP-FA1 (5′-GTCCTGTTCATTGGTACCCG-3′) and 54 MZD d2,3 R (5′-GCTCTAGATTATTTATCATCATCATCTTTATAATCTGGTCGATCACACATGAC-3′) primers were used. The amplified fragments were cloned into the pUASp vector to construct pUASp-s-MZD-FLAG and pUASp-MZDΔ2,3-FLAG, respectively. These vectors were used to generate transgenic flies by standard injection protocols.

### Overexpression of Mamo and Mamo fragments

*matα4-Gal-VP16* flies were crossed with the flies carrying pUASp-Mamo-FLAG, pUASp-MZD-FLAG, pUASp-s-MZD-FLAG and pUASp-MZDΔ2,3-FLAG, respectively. The embryos (Mamo OE) derived from *matα4-Gal-VP16* *>* *UASp-Mamo-FLAG*, the embryos (MamoAF OE) from *matα4-Gal-VP16* *>* *UASp-MZD-FLAG*, the embryos (s-MZD OE) from *matα4-Gal-VP16* *>* *UASp-s-MZD-FLAG*, the embryos (MZDΔ2,3 OE) from *matα4-Gal-VP16* *>* *UASp-MZDΔ2,3-FLAG* were analysed by in situ hybridisation and immunostaining. To investigate the effects of MamoAF and Mamo overexpression on *vas* expression in PGCs, the PGCs in the embryos derived from *nos-Gal4* *>* *UASp-MZD-FLAG* and *nos-Gal4* *>* *UASp-Mamo-FLAG* females were analysed by in situ hybridisation and immunostaining.

### Developmental western blotting of Mamo OE embryos

Mamo OE embryos were collected at 25 °C for 3 h, then the embryos were incubated at 18 °C for 0, 6, 16 and 24 h, respectively. The embryos were dechorionated and homogenised in the sample buffer, and then the samples (40 embryos/lane) were fractionated using a 5–20% gradient sodium dodecyl sulphate-polyacrylamide gel electrophoresis (SDS-PAGE) gel (c520L, Atto) and analysed by western blotting with anti-FLAG antibody.

### Analyses of fractionated Mamo OE and MamoAF OE embryos

Mamo OE and MamoAF OE embryos were collected at 25 °C for 0–16 h. Approximately 0.1 g of the embryos were dechorionated and homogenised using a Dounce tissue grinder in the sucrose solution (0.5 m sucrose, 5 mm HEPES, pH7.9, 5 mm KCl, 1.5 mm MgCl_2_, 0.25 mm DTT, 0.25 mm phenylmethylsulfonyl fluoride (PMSF) supplemented with Protease inhibitor complete EDTA-free protease inhibitor cocktail (Roche)). Debris was removed by centrifugation at 390 × *g* for 10 min at 4 °C and the supernatant was recovered. The supernatant was centrifuged at 6300 × *g* for 10 min at 4 °C. The resulting supernatant was used for cytoplasmic extract, the pellet was suspended in the sucrose solution and used for nuclear extract. Normalised load of each extract was fractionated using a 5–20% gradient SDS-PAGE gel, and then analysed by western blotting with anti-FLAG, anti-Tubulin and anti-Histone H3 antibodies.

### Western blot assays

Samples were denatured in sample buffer and fractionated using SDS-PAGE gel. Then blots were transferred to polyvinylidene difluoride membranes (Immobilon-P, Merck Millipore), and reacted with mouse anti-FLAG M2 antibody (1:2000; Sigma, F1804), anti-V5 antibody (1:5000; abcam, ab27671), anti-tubulin antibody (1:2000; Sigma, T6199) or rabbit anti-histone H3 (1:2000; abcam, ab1791) for 1 h, subsequently with a horseradish peroxidase (HRP)-conjugated mouse IgG antibody (1:2000; Bio-Rad, immune-star anti-mouse HRP) or horseradish peroxidase (HRP)-conjugated rabbit IgG antibody (1:2000; Bio-Rad, immune-star anti-rabbit HRP) for 1 h. Clarity western ECL substrate (Bio-Rad) was used according to the manufacturer’s instructions to detect chemiluminescence. Fluorescent images were obtained using an ImageQuant LAS 4000 system (GE healthcare).

### 5′-RACE and cloning of RACE fragments

Total RNAs were prepared using the Qiagen RNeasy mini kit, according to the manufacturer’s instruction from wild type ovaries. Approximately 1 μg of total RNA was subjected to RNA ligase-mediated 5′-RACE reaction (5′-Full-RACE Core Set) (Takara), according to the manufacturer’s instructions. In brief, the first-strand DNA was synthesised by AMV reverse transcriptase XL using 5′-RACE Short Mamo RT (5′-CCATGTTGAGATCGCC-3′) primer. Inverse PCR was performed with following primer sets. For 1st PCR, 5′-RACE Short Mamo S1 (5′-GCATCAGCAGCAACAGCAG-3′) and 5′-RACE Short Mamo A1 (5′-GATTCGGACGTTTCCGACTC-3′) primer pairs were used. For 2nd PCR, 5′-RACE Short Mamo S2 (5′-GCACATGTTTCCGTGCATC-3′) and 5′-RACE Short Mamo A2 (5′-GATGCCGAACGCTGTTGCATG-3′) primer pairs were used. PCR products were separated on 1% agarose gel, extracted, subcloned into pBSK and sequenced.

### Analyses of germline clones

To investigate the maternal function of CBP, we generated germline clones. We introduced *nej*^*3*^ or *nej*^*0.3*^ mutations into the chromosome carrying FRT (BL1844) by meiotic recombination. The *nej*^*3*^*/Binsinscy* and *nej*^*0.3*^*/Binsinscy* females were mated with *w ovo*^*D1*^ *v*^*24*^ *P{FRT}101;P{hsFLP}38* males, respectively. Their progenies were then subjected to heat shock at 37 °C for 1 h in a water bath. Because the *nej*^*3*^ germline clones were degenerated during mid-oogenesis, we used *CBP*^*M*-^ embryos derived from *nej*^*0.3*^ germline clones were analysed by in situ hybridisation and immunostaining.

### In situ hybridisation to detect Vas mRNA

To detect Vas mRNA in *CBP*^*M*-^ embryos and the embryos homozygous for *vas*^*d10-3*^ at stage 9–10, embryos were collected, dechorionated with sodium hypochlorite for 3 min, then fixed with PBS containing 4% formaldehyde/heptane for 25 min. PBS phase containing formaldehyde was discarded. The vitelline membranes were removed by adding methanol and vigorous shaking. Because *vas*^*d10-3*^was balanced with *CyO, P{w[* *+* *mC]* *=* *GAL4-Kr.C}DC3, P{w[* *+* *mC]* *=* *UAS-GFP.S65T}DC7*, the embryos homozygous for *vas*^*d10-3*^ were selected under the fluorescence stereoscopic microscope. The fixed embryos were hybridised with digoxigenin (DIG)-labelled anti-sense Vas RNA probe in hybridisation solution (50% formamide, 4× SSC, 5% dextransulfate, 0.01% Tween-20) at 55 °C for 16 h^15^, washed with wash solution 1 (50% formamide, 2× SSC, 0.01% Tween-20) for 10 min eight times, then washed with wash solution 1 at 55 °C overnight. Hybridised embryos were rinsed with PBS containing 0.1% Tween-20 (PBTw), then reacted with anti-DIG alkaline phosphatase-conjugated antibody (Roche) at 1:2000 in PBTw containing 5% goat serum for 2 h. The signals were visualised by incubating the embryos with colour developing solution (100 mm Tris-HCl, pH9.5, 100 mm NaCl, 50 mm MgCl_2_, 0.1% Tween-20) containing NBT-BCIP (Roche) at room temperature for 1 h in the dark. To detect Vas mRNA in PGCs of Mamo-OE and MamoAF-OE embryos under the control of maternal *nos-Gal4* driver at stage 14–15, embryos were dechorionated with sodium hypochlorite for 15 s, then fixed with PBS containing 4% paraformaldehyde/heptane for 20 min. The fixed embryos were hybridised with DIG- labelled anti-sense Vas RNA probe in hybridisation solution (50% formamide, 5× SSC, 10% dextransulfate, 0.1% Tween-20, 0.1 mg ml^−1^ yeast RNA, 0.1 mg ml^−1^ heparin, 10 mm DTT) at 60 °C for 16 h^15^, washed with wash solution 2 (50% formamide, 2× SSC, 0.1% Tween-20) at 60 °C for 30 min three times, at room temperature for 10 min three times, and then with wash solution 3 (2× SSC, 0.1% Tween-20) at room temperature for 10 min three times. Hybridised embryos were rinsed with PBTw, then blocked with Blocking reagent (Roche) for 1 h, then reacted with anti-DIG alkaline phosphatase-conjugated antibody (Roche) at 1:3000 for 1 h. To detect Vas mRNA signals, hybridised embryos were incubated with colour developing solution containing NBT-BCIP at room temperature for 30 min in the dark.

### In situ hybridisation to detect the short Mamo mRNA

To make a template for a Mamo BTB/POZ RNA probe, PCR was performed full-length Mamo cDNA as a template using Mamo FL probe Pst F (5′-CAGCTGCAGATGGGCAGTGAG-3′) and Mamo FL probe R (5′-CATGGTGGTGCGTGTGATGG-3′) primers. The resulted PCR product was subcloned and used as a template for producing a Mamo BTB/POZ RNA probe. To make a template for a Mamo Zn-finger RNA probe, PCR was performed MZD-FLAG cDNA^18^ as a template. The resulted PCR product was subcloned and used as a template for producing a Mamo Zn-finger RNA probe. Wild type embryos were collected, dechorionated with sodium hypochlorite for 15 s, then fixed with PBS containing 4% paraformaldehyde/heptane for 20 min. PBS phase containing formaldehyde was discarded. The vitelline membranes were removed by adding methanol and vigorous shaking. In situ hybridisation was performed as described above. To detect Mamo mRNAs encoding Mamo short-isoforms, hybridised embryos were incubated with colour developing solution containing NBT-BCIP at room temperature for 3 h in the dark.

### Double fluorescence in situ hybridisation (double FISH)

To detect ectopic Vas and Ovo mRNA expression in MamoAF-OE embryos, we performed double FISH. To confirm whether ectopic *vas* and *ovo* were expressed in brains, the anti-sense Elav RNA probe, which can specifically detect neural cells^34^, was used. The template for Elav probe was amplified by PCR using Elav probe-F Eco (5′-CGGAATTCGCTAATGCAGAGTGCCGCTG-3′) and Elav probe-R Eco (5′-CGGAATTCCAGACCCTTGTTAACTGGCG-3′) primers from wild type genomic DNA and cloned into pBSK. Fluorescein isothiocyanate (FITC)-labelled anti-sense Elav RNA probe was synthesised from the template using the Fluorescein RNA Labeling Mix (Roche). We used both DIG- and FITC-labelled probes. The DIG-labelled probes for Vas and Ovo mRNAs were visualised by the anti-DIG alkaline phosphatase-conjugated antibody in combination with HNPPD/Fast Red TR (Roche). For FITC-labelled probe for Elav mRNA, the components and protocols of the TSA Plus Fluorescein kit (Perkin Elmer), including the anti-FITC horseradish peroxidase-conjugate antibody and FITC-tyramides as substrates, were used. For double FISH, the embryos were hybridised at the same time using DIG- and FITC-labelled anti-sense RNA probes in hybridisation solution (50% formamide, 5× SSC, 10% dextransulfate, 0.1% Tween-20, 0.1 mg ml^−1^ yeast RNA, 0.1 mg ml^−1^ heparin, 10 mm DTT) at 60 °C for 16 h, washed with solution 2 at 60 °C for 30 min three times, at room temperature for 10 min three times, and then with wash solution three at room temperature for 10 min three times. The embryos were washed with TNT Buffer (0.1 m Tris-HCl, pH7.5, 0.15 m NaCl, 0.05% Tween20) for 10 min three times and blocked with TNB buffer (Perkin Elmer). The embryos were reacted with anti-FITC horseradish peroxidase-conjugate antibody at 1:4000 at 4 °C overnight. The signals of FITC-labelled probe were visualised by incubating the embryos first for 30 min with the amplification solution of TSA kit, and then washed with TNT Buffer (0.1 m Tris-HCl, pH7.5, 0.15 m NaCl, 0.05% Tween20) for 10 min three times. The embryos were incubated with anti-DIG alkaline phosphatase-conjugated antibody at 1:3000 for 1 h. The signals of DIG-labelled probe were visualised by incubating HNPPD/Fast Red TR for 30 min twice. The embryos were mounted with CC/Mount (Diagnostic BioSystems) and imaged with a confocal microscope (LSM 800, Zeiss) within 2 days.

### Rescue experiments

*mamo*^*SVA53*^ and *UASp-Mamo-FLAG* has been described previously^17^. To investigate whether MamoAF can rescue *mamo* mutation, we generated germline clones of *mamo*^*SVA53*^. Unfortunately, the *w ovo*^*D1*^ *v*^*24*^ *P{FRT}101;P{hsFLP}38* strains (BL1813) in both the Bloomington Stock Center and the Genetic Resource Center in Kyoto Institute of Technology lost the *hsFLP* transgene, which is essential to produce germline clones. We crossed the strain with other *hsFLP* strain (Kyoto 108127) to obtain the strain transiently carrying the *hsFLP* transgene. Then, we crossed the strain with *nos-Gal4* to obtain *w ovo*^*D1*^ *v*^*24*^ *P{FRT}101;P{hsFLP}; nos-Gal4* strain. We crossed *w ovo*^*D1*^ *v*^*24*^ *P{FRT}101/Y;P{hsFLP}; nos-Gal4* males with *mamo*^*SVA53*^*/Binsinscy, mamo*^*SVA53*^*/Binsinscy* carrying *UASp-Mamo-FLAG and mamo*^*SVA53*^*/Binsinscy* carrying *UASp-MamoAF-FLAG* females, respectively. Their progenies were then subjected to heat shock at 37 °C for 1 h in a water bath. The resulted females without *Binsinscy* balancer chromosome were crossed with *y w* males to investigate their progenies. The eggs of the females were collected for 3 h. The number of the eggs and their hatching rate were scored. We found that *mamo* phenotype was rescued by expressing Mamo-FLAG. But the phenotype of germline clones was different from that previously reported^17^. We could not obtain the mature eggs derived from germline clones homozygous for *mamo*^*SVA53*^ allele in this experimental condition. This difference is probably owing to *w ovo*^*D1*^ *v*^*24*^ *P{FRT}101; P{hsFLP}38* strain described above. FLP activity may be weaker than original BL1813. Moreover, genetic background of *w ovo*^*D1*^ *v*^*24*^ *P{FRT}101; P{hsFLP}38* strains may affect the efficiency of germline clone production.

### Immunostaining

The embryos were collected and fixed as described above. The fixed embryos were washed with PBS containing 0.1% Triton X-100 (PBTx), blocked PBTx containing 5% goat serum for 1 h and then reacted with primary and secondary antibodies. For primary antibodies, a rabbit anti-Vas antibody at 1:500 from Dr. S. Kobayashi, a mouse anti-Elav monoclonal antibody (Developmental Studies Hybridoma Bank) at 1:100, and a mouse anti-GFP monoclonal antibody (3E6, Thermo Fisher) at 1:300 were used. A mouse anti-H3K27ac monoclonal antibody at 1:500, a mouse anti-H3K9ac monoclonal antibody at 1:500, a mouse anti-H4K16ac monoclonal antibody at 1:500, from Dr. H. Kimura^35^ were used. A mouse anti-Piwi antibody at 1:200 from Dr. M. Shiomi was used to detect PGCs^36^ in Supplementary Fig. 5. AlexaFlour-conjugated antibodies (Molecular Probes) at 1:1000 were used for secondary detection. DNA was stained with DAPI. Stained embryos were mounted with Vectashield (Vector laboratories) and imaged with a confocal microscope (FV2000, Olympus or LSM 800, Zeiss). Fluorescent imaging was acquired with Zen 2.1 Software (Zeiss) or the software for the FV1200 (Olympus).

### Immunostaining of ovaries with *mamo* mutant germline clone

Rescue experiments were performed as described above. To investigate the rescue ability of Mamo and MamoAF, we stained the ovaries containing *mamo* mutant germline clone expressing full-length Mamo-FLAG or MamoAF-FLAG. Germline clones were generated as described above. Females carrying *mamo* mutant germline clones were dissected. The ovaries were fixed with PBS containing 4% paraformaldehyde for 20 min. The ovaries were washed with PBS containing 0.1% Triton X-100 (PBTx), blocked PBTx containing 5% goat serum for 1 h and then reacted with primary and secondary antibodies. For primary antibodies, a rabbit anti-Vas antibody at 1:500. AlexaFlour-conjugated antibodies (Molecular Probes) at 1:1000 were used for secondary detection. DNA was stained with DAPI. Stained ovaries were mounted with Vectashield (Vector laboratories) and imaged with a confocal microscope (FV2000, Olympus). Fluorescent imaging was acquired with the software for the FV1200 (Olympus).

### Immunostaining of ovaries overexpressing Mamo

Ovaries were dissected from females of *nos-Gal4, UASp-Mamo-FLAG* and *nos-Gal4, UASp-MamoAF-FLAG*. The ovaries were fixed with PBS containing 4% paraformaldehyde for 20 min. The ovaries were washed with PBS containing 0.5% Triton X-100 (PBTx0.5), blocked PBTx0.5 containing 5% goat serum for 1 h and then reacted with primary and secondary antibodies. For primary antibodies, a rabbit anti-Vas antibody at 1:500, a mouse anti-FLAG monoclonal antibody M2 (Sigma) at 1:200 were used. AlexaFlour-conjugated antibodies (Molecular Probes) at 1:1000 were used for secondary detection. DNA was stained with DAPI. Stained ovaries were mounted with Vectashield (Vector laboratories) and imaged with a confocal microscope (LSM 800, Zeiss). Fluorescent imaging was acquired with Zen 2.1 Software (Zeiss).

### Detection of ectopic Vas expression

Ectopic Vas expression was detected in heterogenous cell population in embryonic brain and VNC. To gain the image, a single section image was used. Ectopic Vas signals were normalised by DAPI signals.

### RNA preparation and qRT-PCR

Total RNAs were prepared using the Qiagen RNeasy mini kit according to the manufacturer’s instructions from wild-type embryos, Mamo OE, MamoAF OE, CBP OE, and the embryos overexpressed both CBP and MamoAF. RNAs were reverse-transcribed using SuperScript III. Expression levels of *vas* were quantified using the SYBR Premix Ex TaqTM II (Tli RNaseH Plus) (Takara) using standard curve method. Expression levels of *vas* were normalised to *Rp49* expression. Following primers were used: the RT-rp49F (5′-AGCGCACCAAGCACTTCATC-3′) and Rt-rp49 R (5′-GACGCACTCTGTTGTCGATACC-3′) primers for *Rp49*, the RT-vas sense (5′-TTGCGTTGCGCGAAGTGAT-3′) and RT-vas anti-sense (5′-CGCCGCGAGTATCAACAAT-3′) primers for *vas*. Expression levels of *vas* were quantified using the software for Thermal Cycler Dice TP700 (Takara).

### EMSA

Bacterially expressed GST-MZD and GST proteins were purified^18^. *Escherichia coli* BL21 transformed with the GST-MZD bacterial expression vector, pGEX GST-MZD^18^, was grown at 37 °C to OD600 = ~ 0.5 and then induced by the addition of IPTG to a final concentration of 0.5 mm. The cells were grown for an additional 120 min at 37 °C. The cells were collected by centrifugation, sonicated in the sonication buffer (2 mm Na_2_HPO_4_, 0.2 mm KH_2_PO_4_, 13.7 mm NaCl containing 1 mm DTT, 0.5 mm PMSF, 0.1% Triton X-100) and affinity-purified by Glutathione-Sepharose 4B (GE Healthcare). Eluted proteins were dialysed against the dialysis buffer (20 mm HEPES, pH 7.9, 50 mm KCl, 2 mm MgCl_2_, 10% glycerol, 1 mm DTT, 1 mm PMSF) three times for 2 h at 4 °C. The purified proteins were concentrated by Microcon (Millipore). Sequences of the DNA probes were as follows: *vas*-A (5′-ATTTGTTTTTCCTGCGTTGCCTGCTGAGT-3′); *vas*-AΔ, (5′-ATTTGTTTTTCCTGCCTGCTGAGT-3′). Sense and anti-sense oligonucleotides for each probe were annealed, DIG-labelled and subjected to EMSA assay using the DIG Gel shift Kit 2nd Generation (Roche). Purified GST-MZD or GST protein (60 ng) and DIG-labelled probe (30 fmol) were incubated in the binding buffer (20 mm HEPES, pH 7.6, 1 mm EDTA, 10 mm (NH_4_)_2_SO_4_, 1 mm DTT, 0.2% Tween20, 30 mm KCl, 1 μg poly[d(I-C)], 0.1 μg poly l-lysine) for 15 min at 25 °C. Binding reactions were electrophoresed in 7% polyacrylamide gels, and transferred to a positively charged nylon membrane (Roche). The membrane was then UV cross-linked, blocked and probed with an anti-DIG Fab fragment (Roche). DIG-labelled DNA–protein complexes were detected by CSPD (Roche). Chemiluminescence images were obtained using an ImageQuant LAS 4000 system (GE healthcare).

### Mutagenesis using CRISPR/Cas9 system

Guide RNAs (gRNAs) were designed (Supplementary Fig. 8), synthesised and purified using the SmartNuclease TM T7 gRNA vector (System Biosciences) according to the manufacturer’s instructions. gRNA was injected into the embryos derived from *y*^*2*^ *cho*^*2*^ *v*^*1*^*; attP40{nos-Cas9}/CyO* females by standard injection protocols. Target sequences were amplified by PCR, annealed and the resulted heteroduplexes were digested with T7 endonuclease I (NEB) to detect Cas9-induced mutations. Following primers were used: CRISPR vas Fv2 (5′-CAAACACCGAACGAATTCC-3′) and CRISPR vasA R (5′-TTCCGATAGAACTGGTGAG-3′). Deletions were confirmed by sequencing (accession No. LC503775 at DNA Data Bank of Japan).

### ChIP analyses

Dechorionized wild type embryos and MamoAF-OE (~ 0.15 g) were homogenised with Dounce tissue grinder in the buffer (15 mm HEPES, pH 7.5, 15 mm NaCl, 60 mm KCl, 4 mm MgCl_2_, 0.5% Triton X-100, 0.5 mm DTT, complete EDTA-free protease inhibitor cocktail (Roche)) containing 0.8% formaldehyde on ice and then were fixed for 15 min at room temperature. The fixed embryos were recovered by centrifugation at 1430 x *g* for 5 min at 4 °C, and then washed with the buffer (15 mm HEPES, pH 7.5, 140 mm NaCl, 1 mm EDTA, 0.5 mm EGTA, 1% Triton-X-100, 0.1% sodium deoxycholate, 0.1% SDS, 0.5% *N*-lauroylsarcosine, supplemented with complete EDTA-free protease inhibitor cocktail (Roche)). The fixed embryos were sonicated with a Branson Model Sonifier 250 sonicator using a microtip, and then centrifuged at 15,880 x *g* for 30 min at 4 °C to recover the supernatant containing fragmented chromatin. Dynabeads M-280 sheep anti-mouse IgG (Thermo Fisher), mouse anti-FLAG monoclonal antibody M2 (Sigma) and mouse anti-H3K27ac monoclonal antibody^35^ were used for chromatin immunoprecipitation. Input DNA, mock-precipitated DNA with control mouse IgG and DNA from the immunoprecipitation were analysed by PCR. Quantitative PCR (qPCR) analyses were performed using SYBR Premix Ex TaqTM II (Tli RNaseH Plus) (Takara). Following primers were used: vasa A chip F (5′- GTAATTGGTTACGCGTAAAC-3′) and vasa A chip R (5′-AACGAGCGCGCCCATAC-3′).

### S2 cell culture

*Drosophila* S2 cells were cultured in Schneider’s *Drosophila* medium (Gibco) supplemented with 10% heat-inactivated fetal bovine serum (Biosera).

### Expression vectors for coimmunoprecipitation assays

The expression vectors of MamoAF and OvoB used in coimmunoprecipitation assays was constructed as follows. MamoAF fragment was amplified from the pUASp-MZD-FLAG plasmid^18^ by PCR. Eco-MZD-puastV2 (5′-CGGAATTCATGGATGCCATGCCCGTGATT-3′) and puast-MZD-R-EcoR1 (5′-GGAATTCTTATTTATCATCATCATCTTTAT-3′) primers were used. The amplified fragment was cloned into the pUASt vector to construct pUASt-MamoAF-FLAG. The coding sequence of OvoB was amplified from pAC-OvoB by PCR and cloned into the pUASt vector to construct pUASt-OvoB-V5. The expression vectors of MamoAF fragments used in coimmunoprecipitation assays (Supplementary Fig. 14) was constructed as follows. MamoAF fragments were amplified from the pUASt-MamoAF-FLAG plasmid by PCR. For s-MamoAF(251–550)-FLAG, s-MamoAF-1-SacIIF (5′-CTACCGCGGATGCCGCTGCACATGTTTCCG-3′) and MamoAF-R-Xba (5′- CGCGTCTAGATTATTTATCATCATCATCTTT-3′) primers were used. For s-MamoAF(278–550)-FLAG, s-MamoAF-2-SacIIF (5′- CTACCGCGGATGTCGCTGAAGACGCTCGAG-3′) and MamoAF-R-Xba (5′-CGCGTCTAGATTATTTATCATCATCATCTTT-3′) primers were used. For s-MamoAF(454–550)-FLAG, s-MamoAF-3-SacIIF (5′-CTACCGCGGATGGCCGCGTCCAGTGATCC-3′) and MamoAF-R-Xba (5′- CGCGTCTAGATTATTTATCATCATCATCTTT-3′) primers were used. The amplified fragments were cloned into the pUASt vector, respectively. The expression vectors of OvoB fragments used in coimmunoprecipitation assays (Supplementary Fig. 15) was constructed as follows. pUASt-OvoB-V5 plasmid was digested with *Sac*II and the resulted fragment was removed and self-ligated to construct pUASt- OvoB(338–982)-V5. pUASt-OvoB-V5 plasmid was digested with *Not*I and the resulted fragment was removed and self-ligated to construct pUASt- OvoB(470–982)-V5. pUASt-OvoB-V5 plasmid was digested with *Eco*RI and the resulted fragment was removed and self-ligated to construct pUASt- OvoB(758–982)-V5.

### Coimmunoprecipitation assays

In the coimmunoprecipitation assays for MamoAF and OvoB, both proteins were overexpressed in S2 cells. Approximately 1.4 μg of pWAGal4 (a gift from Dr. Y. Hiromi), 4 μg of pUASt-MamoAF-FLAG and 4 μg of pUASt-OvoB-V5 plasmid vectors were transfected into S2 cells (3.6 × 10^7^) using Effectene transfection reagent (Qiagen) and then cultured in the culture medium supplemented with Penicillin–Streptomycin–Amphotericin B Suspension (Wako) for 3 days. The transfected S2 cells were incubated in the buffer (10 mm HEPES pH7.5, 10 mm KCl, 0.1 mm EDTA, 0.4% NP-40, supplemented with Protease inhibitor complete EDTA-free protease inhibitor cocktail) for 10 min on ice, and then were centrifuged to obtain the nuclear fraction. Nuclear extracts were prepared from the nuclear fraction by incubating in the nuclear lysis buffer (50 mm HEPES pH7.5, 3 mm MgCl_2_, 300 mm NaCl, 1 mm DTT, 0.1 mm PMSF, Protease inhibitor complete EDTA-free) for 2 h at 4 °C with shaking, then incubated with 8 μg ml^−1^ DNase, 8 μg ml^−1^ RNase and 40 U ml^−1^ Benzonase (Merck) at 4 °C for 30 min. The nuclear extracts were incubated with 2 μg of rabbit anti-V5 antibody (Abcam, ab9116) or 2 μg of rabbit anti-FLAG antibody (Sigma, F7425) or 2 μg of rabbit control IgG (Cell signalling technology, 2729 S) in the nuclear lysis buffer for 2 h at 4 °C. The immunocomplexes were precipitated with Dynabeads M-280 sheep anti-rabbit IgG (Thermo Fisher) and washed with the ice-cold buffer (50 mm Tris-HCl, pH7.5, 0.3 m NaCl, 10% glycerol, 0.1% NP-40) using DynaMagTM-2. Finally, the complexes were fractionated using 10% PAGE gel, and analysed by western blotting.

### Expression vectors for reporter assays

MamoAF fragment was amplified from the pUASp-MZD-FLAG^18^ plasmids by PCR. The amplified fragment was cloned into the pAC vector. The coding sequences of OvoB^16^ and CBP, a gift from Dr. M. Mannervik, were cloned into the pAC vector to construct pAC-OvoB and pAC-CBP, respectively.

### Luciferase reporter constructs

The genomic fragment of the first intron of *vas* (2 L: 15,061,805–15,068,333) was amplified by PCR and cloned into pBSK to obtain pBSK-vas 1st intron plasmid. Following the primers, vas 1st intron F2 H3 (5′-CCCAAGCTTAGTGATATGCAATTAGTTTT-3′) and vas 1st intron R2 H3 (5′-CCCAAGCTTAATACGTTCATTTCTAAAGT-3′) were used for PCR. pBSK-vas 1st intron plasmid was digested with restriction enzymes to produce the genomic fragments containing the cis-elements in the first intron of *vas*, Ovo1, Ovo2, *vas*-A and Ovo2 + *vas*-A fragments (Supplementary Fig. 11). These fragments were cloned into a luciferase reporter enhancer assay vector (Promega, pGL4.23), respectively. *vas*-A and Ovo1 Sac I fragments were amplified by PCR. For Ovo1 Sac I fragment, F Ovo1 up250-SacI(5′-GGATGAGCTCGAAAGTTTCCCTCCAAC-3′) and R Ovo1 down250-SacI(5′- CTTTGAGCTCAAGAAAAATACACTATTAC-3′) primers were used. For *vas*-A fragment, PstI-vas intron A up (5′-TGCACTGCAGATGTTCTGTCCGACCATTAG-3′) and vas intron A down500 (5′-TGCACTGCAGGTCGGAGCACCCGATGG-3′) primers were used. These fragments were ligated with linkers and cloned into pGL4.23 to construct Ovo1 + *vas*-A luc reporter construct. The PCR-based, site-directed mutagenesis procedure was used to produce Ovo1mut and *vas*-A mut fragments (Fig. 6 and Supplementary Fig. 11). For Ovo1mut, vas 1st intron ovo bind seq1 (5′-CAGGGATCTTCAAATTAGCG-3′) and 1R-Ovo1-del-sma1 (5′-CCTTTCCCGGGGTGTTTCTTGGATGCAAA-3′) primers were used for first round of PCR. 2F-ovo1-del-sma1 (5′-AACACCCCGGGAAGGTTTTAACACTTAC-3′) and 2R-ovo1-down700nde1 (5′-AAATACCAACCAACAAGCTAAGTAA-3′) primers were used second round of PCR reaction. For *vas*-A mut, 1 F StuIvasA 750up (5′-CAATGATTATGCCTGCAAAGGCCTC-3′) and 1R3 vasA MZD bind-afl2 (5′-CAGGCACTTAAGGAAAAACAAATTCCATC-3′) primers were used for first round of PCR. 2F3 vasA MZD bind-afl2 (5′-TTTTCCTTAAGTGCCTGCTGAGTTTGATT-3′) and 2 R vasA900down PstI (5′-CCATTAGATAATCTTGTGGCGCCGAC-3′) primers were used for second round of PCR reaction.

### Luciferase reporter assays

The luciferase reporter constructs containing the cis-elements in the first intron of *vas* described above were used for luciferase reporter assays. The hRluc/TK (Promega, pGL4.74) served as an internal control. About 50 ng of a luc reporter construct and 1 ng of an internal control were co-transfected into S2 cells (3 × 10^4^ cells) with either 10 ng of pAC-MamoAF-FLAG and/or 1 ng of pAC-OvoB and/or 1 ng of pAC-CBP and/or pBSK (for mocked transfected cells) using Effectene transfection reagent (Qiagen). The transfected cells were cultured in the culture medium supplemented with Penicillin–Streptomycin–Amphotericin B Suspension (Wako). Cells were lysed after 1 day of transfection with a passive lysis buffer (Promega) and luciferase activity was measured using a Dual-Luciferase reporter assay system (Promega). All experiments were carried out in triplicate.

### Statistics and reproducibility

Statistical tests were employed for each experiment are indicated in the figure legends. Microsoft Excel was used for statistical analyses. For all data in these analyses, *P* values of < 0.05 were considered to be significant. Replicate experiments were successful.

### Reporting summary

Further information on experiments and research design are available in the Nature Research Reporting Summary linked to this article.

## Supplementary information

Supplementary Information

Description of Additional Supplementary Files

Supplementary Data 1

Reporting Summary

## Data Availability

The data sets generated during and/or analysed during the current study are available from the corresponding author on reasonable request. The source data underlying plots are provided in Supplementary Data 1. Full blots are shown in Supplementary Information. The sequencing data confirming *vas* mutants were deposited into the DNA Data Bank of Japan under the accession number LC503775.
