# Supplementary material for: A truncated form of a transcription factor Mamo activates vasa in Drosophila embryos
Source: Commun Biol. 2019 Nov 20;2:422. doi: 10.1038/s42003-019-0663-4 (PMC6868150; doi:10.1038/s42003-019-0663-4)
Supplement: Supplementary file 1 — Supplementary Information [file 42003_2019_663_MOESM1_ESM.pdf]

```
1    atgggcagtgagcactactgcttgaggtggaacaattaccaatccaacctgttaggggtgtttagtcaattactacaagacgggagcctggtggacgtcacactagtttgttcagagggc 120
1    M  G  S  E  H  Y  C  L  R  W  N  N  Y  Q  S  N  L  L  G  V  F  S  Q  L  L  Q  D  G  S  L  V  D  V  T  L  V  C  S  E  G  40

121  acatccatcagagcccataaggtggtcctctcggcttgttcctcgtacttccagagcctgttcctggagcaccccgaaggacatctaattgtgatattgaaggacgtccgttttgccgag 240
41   T  S  I  R  A  H  K  V  V  L  S  A  C  S  S  Y  F  Q  S  L  F  L  E  H  P  E  G  H  L  I  V  I  L  K  D  V  R  F  A  E  80

241  ctgcaaaccttagttgaattcatgtacaagggcgaggtgaacgtgcaatattgtcagttgtccgcgctgctcaagacagccgaatccctcaaggtcaagggcctggccgagatgacgaac 360
81   L  Q  T  L  V  E  F  M  Y  K  G  E  V  N  V  Q  Y  C  Q  L  S  A  L  L  K  T  A  E  S  L  K  V  K  G  L  A  E  M  T  N  120

361  cagaatacgacgctgcgcgaaccggaacgggaaccggatcgcttgcgtccgcagggcggcggcggtagcggcggatccgcccacaaggcggccgacagtccggcggccgcgcttgatgcc 480
121  Q  N  T  T  L  R  E  P  E  R  E  P  D  R  L  R  P  Q  G  G  G  G  S  G  G  S  A  H  K  A  A  D  S  P  A  A  A  L  D  A  160

481  acagcggccacacaggcggcgactgccacggcaacggcaactgccgcggcggcaacatcgacctcaacctcagcaacatctgcggcggcgacggcagcagcgacagcagcaacatctgcg 600
161  T  A  A  T  Q  A  A  T  A  T  A  T  A  T  A  A  A  A  T  S  T  S  T  S  A  T  S  A  A  A  T  A  A  A  T  A  A  T  S  A  200

601  tccacaacggcaacggcggcggcggggagtagtaatacaacaactacaacggcggcaaccacaacagcaacctgtgccacagcagcaacatctacggcagcaacatcctcatcttcgtcc 720
201  S  T  T  A  T  A  A  A  G  S  S  N  T  T  T  T  T  A  A  T  T  T  A  T  C  A  T  A  A  T  S  T  A  A  T  S  S  S  S  S  240

721  gtgacatcagcagcagcagccgcagcagcagccgcatcagcatctggtgttgcgcactcggaggaggcgacctcgtcatcatcgtcaactggcgggcagaagcgggaggctagcgatcgc 840
241  V  T  S  A  A  A  A  A  A  A  A  A  S  A  S  G  V  A  H  S  E  E  A  T  S  S  S  S  S  T  G  G  Q  K  R  E  A  S  D  R  280

841  agcagtcccacgcccgccaaacgatcgtcgcgaatgcatccggcggacaaggtggatgtggcagaggatcgggaacaggagcaaaaccaggcggacgagctgctaagcgaggagctgctc 960
281  S  S  P  T  P  A  K  R  S  S  R  M  H  P  A  D  K  V  D  V  A  E  D  R  E  Q  E  Q  N  Q  A  D  E  L  L  S  E  E  L  L  320

961  ggtcgcaacttaaaggacgaggaggatgacgatgtggatgagctagatgagaacgaggagacgaagctgaggggtcgcgatgaggacgatgacgacgaggatgaggaggaggacacgccc 1080
321  G  R  N  L  K  D  E  E  D  D  D  V  D  E  L  D  E  N  E  E  T  K  L  R  G  R  D  E  D  D  D  D  E  D  E  E  E  D  T  P  380

1081 atgccattggatctctatcaaagaccaaatgtcgagccaaaaagcgccgcagcagcaacagcagcgcagcagggcggcagcaacagtctcagcggtagcagcaacgcttgccacaccaac 1200
361  M  P  L  D  L  Y  Q  R  P  N  V  E  P  K  S  A  A  A  A  T  A  A  Q  Q  G  G  S  N  S  L  S  G  S  S  N  A  C  H  T  N  400

1201 agcagcagcaacagcaataataacaataacaacaacaacaactccagcagcaacaacaacaacaacactagtagtgggaaaacctcagcgtccagcaatggcggcacagcatcatcgggc 1320
401  S  S  S  N  S  N  N  N  N  N  N  N  N  N  S  S  S  N  N  N  N  N  T  S  S  G  K  T  S  A  S  S  N  G  G  T  A  S  S  G  440

1321 ggcacagcggactccgtggttgcggtagatgacgatgacgacgacgacgatggtggccatcatcatcagcgacaggtgatggacgatcgattggagcaggatgtggacgaagaggatctg 1440
441  G  T  A  D  S  V  V  A  V  D  D  D  D  D  D  D  D  G  G  H  H  H  Q  R  Q  V  M  D  D  R  L  E  Q  D  V  D  E  E  D  L  480

1441 gacgatgatgtcgtggtggtgggacctgccaccgcgatggccaggggcattgcccagcggctggcccaccagaacctgcagcgtctccatcacacgcaccaccatgcccagcaccagcat 1560
481  D  D  D  V  V  V  V  G  P  A  T  A  M  A  R  G  I  A  Q  R  L  A  H  Q  N  L  Q  R  L  H  H  T  H  H  H  A  Q  H  Q  H  520

1561 tcgcagcaccaccacccacattcgcagcaccaccatacgccgcaccaccagcagcaccatacgcactcggatgacgaggatgccatgcccgtgattgccaagagcgagatcctcgacgat 1680
521  S  Q  H  H  H  P  H  S  Q  H  H  H  T  P  H  H  Q  Q  H  H  T  H  S  D  D  E  D  A  M  P  V  I  A  K  S  E  I  L  D  D  560

1681 gactacgacgatgagatggatctggaggatgacgatgaggcggacaacagcagcaacgatttgggtctcaacatgaaaatgggcagcggcggtgcgggcggcggcggtggcgtagatctg 1800
561  D  Y  D  D  E  M  D  L  E  D  D  D  E  A  D  N  S  S  N  D  L  G  L  N  M  K  M  G  S  G  G  A  G  G  G  G  G  V  D  L  600
                                                                                        ESTs cDNA clone
1801 tccaccggctccacattgataccctcgccattgatcaccctgccatcctcatcggcggcggcggcggcagctgcagcggcagcaatggaatcgcagcgctccacgccggccatgtcggcg 1920
601  S  T  G  S  T  L  I  P  S  P  L  I  T  L  P  S  S  S  A  A  A  A  A  A  A  A  A  A  M  E  S  Q  R  S  T  P  A  M  S  A  640
                                                                                                              *1 *2 5' RACE clones
1921 gcccaggcagctggagcgggcgcctcggacctcagtattggtggctgcggcggtcgaccggcgtccaggagctcacgcgacggcggcggtaacgatggcggcaggggaggagcatcctcc 2040
641  A  Q  A  A  G  A  G  A  S  D  L  S  I  G  G  C  G  G  R  P  A  S  R  S  S  R  D  G  G  G  N  D  G  G  R  G  G  A  S  S  680

2041 tccagcctgcttagcccgggaaccgtggccaatctcctgcccgtgcaatcggtgccattgagtctcaagaaggagatcgattgcagcgaggacgacaatagcagtcacagcagacacatg 2160
681  S  S  L  L  S  P  G  T  V  A  N  L  L  P  V  Q  S  V  P  L  S  L  K  K  E  I  D  C  S  E  D  D  N  S  S  H  S  R  H  M  720

2161 caacagcgttcggcatcagctggcggcggcctgggcggcgaccttgactaccgcgcctcccacgagtcggaaacgtccgaatcgccccaccaacaacaacagccccccaccacacgctgtg 2280
721  Q  Q  R  S  A  S  A  G  G  G  L  G  G  D  L  D  Y  R  A  S  H  E  S  E  T  S  E  S  P  H  Q  Q  Q  Q  P  P  P  H  A  V  760

2281 ggcgttggggcgggcggtggctcagcggccgcagcagctgccgcagcagccgccctgctgcatcagcagcaacagcagcagcccccacacagcccccccgccgccgccactgcacatgttt 2400
761  G  V  G  A  G  G  G  S  A  A  A  A  A  A  A  A  A  A  L  L  H  Q  Q  Q  Q  Q  Q  P  P  H  S  P  P  P  P  P  L  H  M  F  800

2401 ccgtgcatctactgccgctcggccttccccaaccaatcgaaactcacccgccacctgctgacgcactcgctgaagacgctcgagtatcgcgaaccaaccaccacggctctgctccattcg 2520
801  P  C  I  Y  C  R  S  A  F  P  N  Q  S  K  L  T  R  H  L  L  T  H  S  L  K  T  L  E  Y  R  E  P  T  T  T  A  L  L  H  S  840

2521 catctgctgggcgatctcaacatggccgccgccggcctaccatcgccattcgcctttcagatgaacgccgccgcaatggcggccacttccggtagcgatacacaggagcaggtggcggca 2640
841  H  L  L  G  D  L  N  M  A  A  A  G  L  P  S  P  F  A  F  Q  M  N  A  A  A  M  A  A  T  S  G  S  D  T  Q  E  Q  V  A  A  880

2641 ttggccgccgcctttggcatcgatatggaggcggccctctcgttggccagcaccggctgtacgttccagagtttgactgccagctgcctggaggcaagctccggcggcggaagcggtggc 2760
881  L  A  A  A  F  G  I  D  M  E  A  A  L  S  L  A  S  T  G  C  T  F  Q  S  L  T  A  S  C  L  E  A  S  S  G  G  G  S  G  G  920

2761 ggcggccagggttcgttgtcccgctcggcaacttcagccggaaacggtggtggcctaatgctcggcagcggaagtggcagtggctcgggggctgcagcggcaacgggtggcagcggcagc 2880
921  G  G  Q  G  S  L  S  R  S  A  T  S  A  G  N  G  G  G  L  M  L  G  S  G  S  G  S  G  S  G  A  A  A  A  T  G  G  S  G  S  960

2881 agcggctgtggtggcggtggcggtaccgccacatccggctccttgtcgccatccacatccgccgcagcagcagcggcggccgctgcacaatcgcagtcagcggcgctgctgggcgccgcg 3000
961  S  G  C  G  G  G  G  G  T  A  T  S  G  S  L  S  P  S  T  S  A  A  A  A  A  A  A  A  A  Q  S  Q  S  A  A  L  L  G  A  A  1000

3001 tccagtgatccgaatagtgttgtcatgtgtgatcgaccatttaaatgtgagttttgtggcaaagcatttaagctgcgccaccacatgaaagatcattgtcgcgtgcacaccggcgaacgg 3120
1001 S  S  D  P  N  S  V  V  M  C  D  R  P  F  K  C  E  F  C  G  K  A  F  K  L  R  H  H  M  K  D  H  C  R  V  H  T  G  E  R  1040

3121 ccgttcagatgtaatatgtgcgggaaaactttctcgcgctcaacgatactcaaggcgcacgagaaaacgcatttcccaagtacgtgcgcaagtttttgtcacccagcagcccggtcgat 3240
1041 P  F  R  C  N  M  C  G  K  T  F  S  R  S  T  I  L  K  A  H  E  K  T  H  F  P  K  Y  V  R  K  F  L  S  P  S  S  P  V  D  1080

3241 acaaaggatgagttgccgcaatag 3264
1081 T  K  D  E  L  P  Q  *  1087
```

Supplementary Figure 1

Nucleotide and predicted amino acid sequences of full-length and short-form Mamo cDNAs. Arrows indicate the transcription initiation site of EST clones (GM29022 and GM29051), and the transcription initiation sites revealed by 5’-RACE, respectively. The alternative transcriptional start sites indicated by the arrows labeled with #1 and #2. The number of clones isolated with each transcription initiation sites are 5 and 2, respectively. Boxes indicates translational start codon of short-isoforms of Mamo, respectively. Grey indicates the BTB/POZ-domain. Yellow indicate the Zn-finger domains.

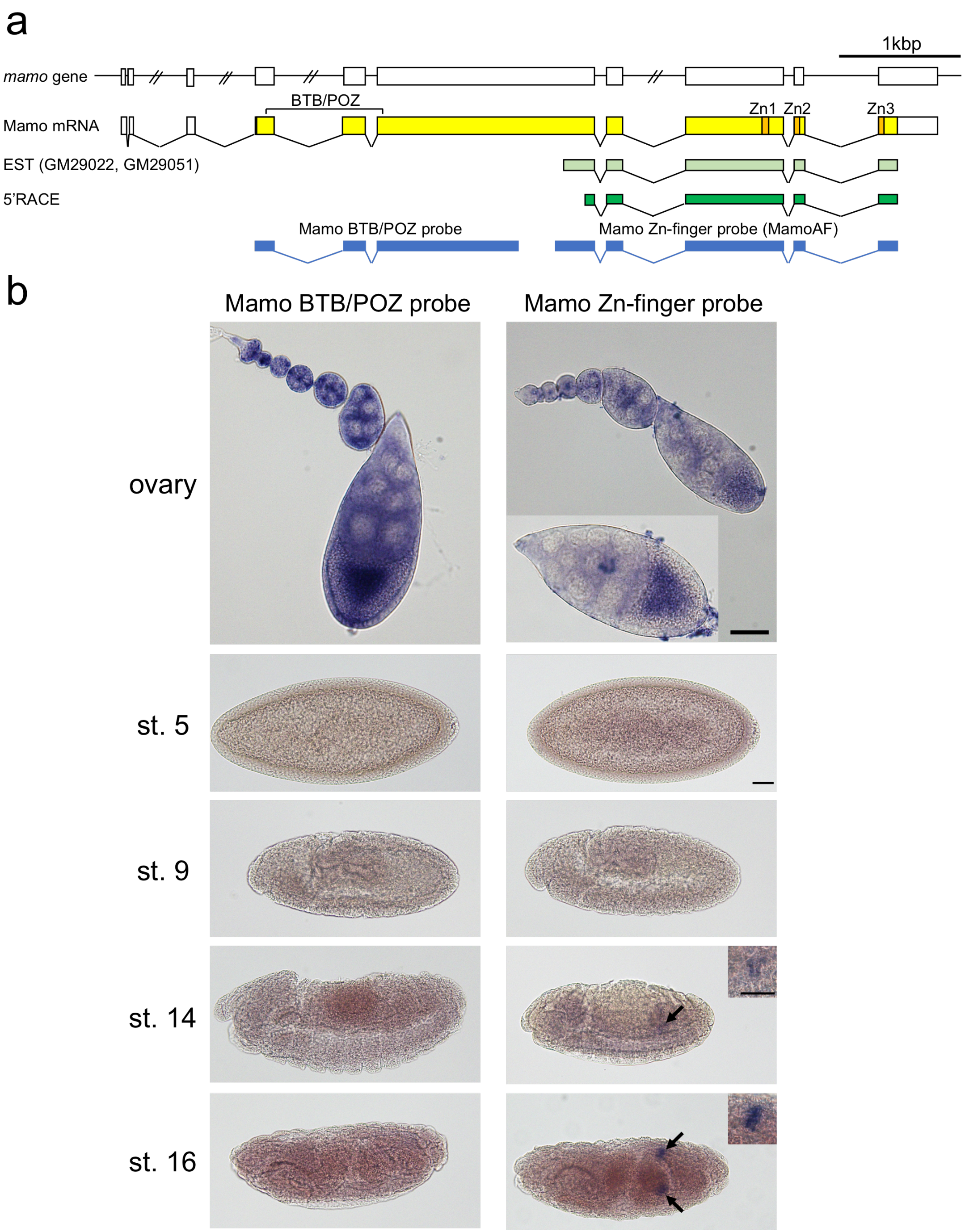
a
1kbp
mamo gene
BTB/POZ
Mamo mRNA
Zn1
Zn2
Zn3
EST (GM29022, GM29051)
5'RACE
Mamo BTB/POZ probe
Mamo Zn-finger probe (MamoAF)
b
Mamo BTB/POZ probe
Mamo Zn-finger probe
ovary
st. 5
st. 9
st. 14
st. 16

Supplementary Figure 2

The Mamo mRNA encoding Mamo short-isoform is expressed in PGCs. **a** A schematic diagram of *mamo* locus. **b** *in situ* hybridisation of wild type ovaries and embryos using a Mamo BTB/POZ probe (left panels) or a Mamo Zn-finger probe (right panels). Arrows indicate the embryonic gonads containing PGCs, in which Mamo mRNA encoding Mamo short-isoform is expressed. Insets indicate close-up views of the embryonic gonads. Scale bar: 30 μm.

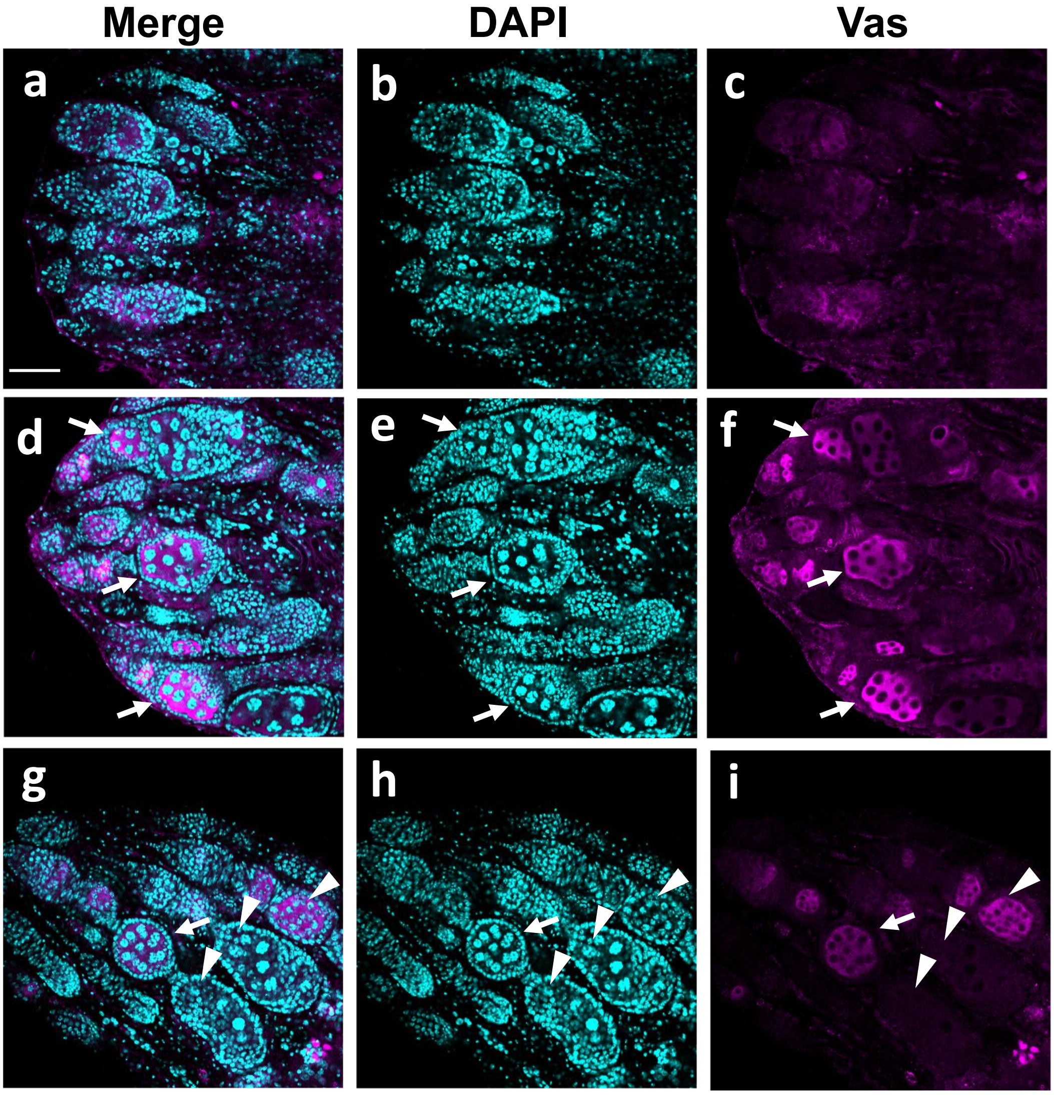
Merge
DAPI
Vas
a
b
c
d
e
f
g
h
i

Supplementary Figure 3 Differentiation of egg chambers containing *mamo* mutant germline clones expressing full-length Mamo-FLAG or MamoAF-FLAG. Immunostaining of the ovarioles containing *mamo* mutant germline (**a**-**c**) and the ovarioles containing *mamo* mutant germline clones expressing full-length Mamo-FLAG (**d**-**f**) and MamoAF-FLAG (**g**-**i**). Arrows indicate differentiating egg chambers after oogenic stage 6. Arrowheads indicate degenerating egg chambers. Scale bar: 50 μm.

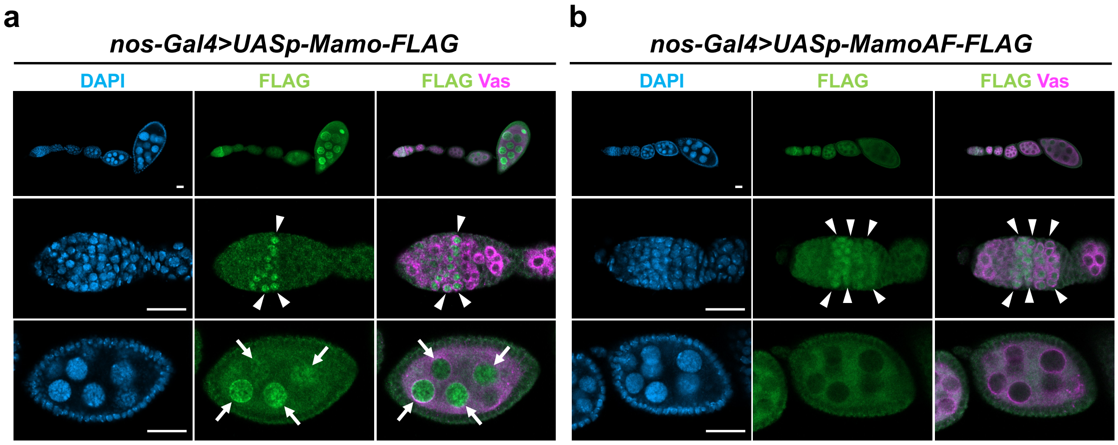
a
nos-Gal4>UASp-Mamo-FLAG
DAPI
FLAG
FLAG Vas
b
nos-Gal4>UASp-MamoAF-FLAG
DAPI
FLAG
FLAG Vas

Supplementary Figure 4 Expression patterns of full-length Mamo-FLAG and MamoAF-FLAG in ovaries. Immunostaining of the ovarioles expressing full-length Mamo-FLAG (**a**) or MamoAF-FLAG (**b**) under the control of *nos-Gal4* driver. Arrowheads indicate the signals of full-length Mamo-FLAG or MamoAF-FLAG in the nuclei of germline cysts in the germarium. Arrows indicate the full-length Mamo-FLAG signals in the nuclei of nurse cells in the egg chamber at stage 6. Scale bar: 20 μm.

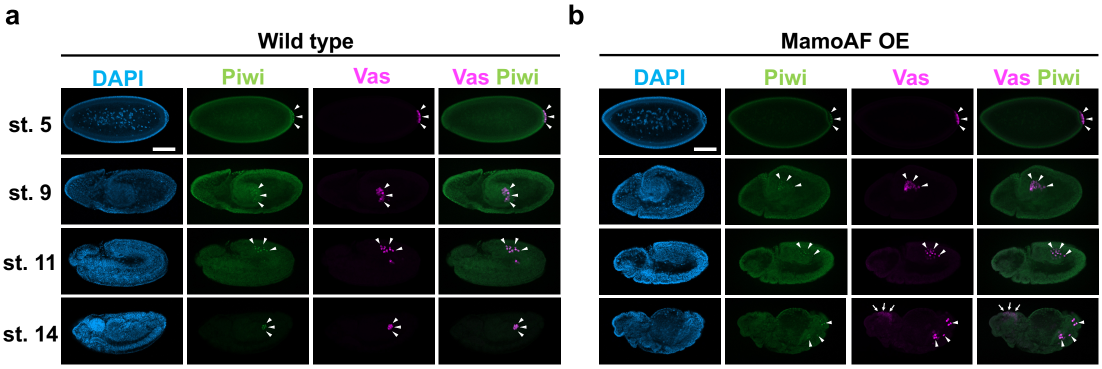

a
Wild type
DAPI
Piwi
Vas
Vas Piwi
st. 5
st. 9
st. 11
st. 14
b
MamoAF OE
DAPI
Piwi
Vas
Vas Piwi

Supplementary Figure 5 PGC formation and migration in MamoAF-OE embryos. Immunostaining of wild type (**a**) and MamoAF OE embryos (**b**). Arrowheads indicate PGCs. Arrows indicate ectopic Vas signals. Piwi was used as a PGC marker. Maternal Piwi is enriched and maintained in PGCs during embryogenesis. Scale bar: 100 μm.

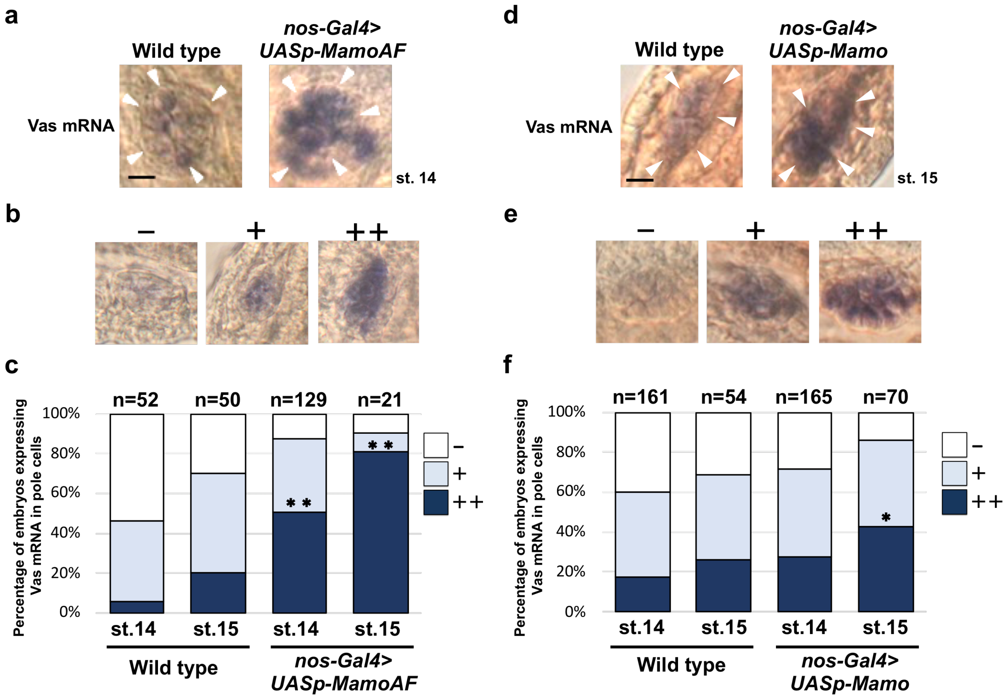

a
Wild type
nos-Gal4>
UASp-MamoAF
Vas mRNA
st. 14
b
−
+
++
c
Percentage of embryos expressing Vas mRNA in pole cells
n=52
n=50
n=129
n=21
100%
80%
60%
40%
20%
0%
st.14
st.15
st.14
st.15
Wild type
nos-Gal4>
UASp-MamoAF
**
**
−
+
++
d
Wild type
nos-Gal4>
UASp-Mamo
Vas mRNA
st. 15
e
−
+
++
f
Percentage of embryos expressing Vas mRNA in pole cells
n=161
n=54
n=165
n=70
100%
80%
60%
40%
20%
0%
st.14
st.15
st.14
st.15
Wild type
nos-Gal4>
UASp-Mamo
*
−
+
++

Supplementary Figure 6 MamoAF and Mamo overexpression promote Vas mRNA expression in PGCs. **a** Vas mRNA *in situ* hybridisation of wild type and the embryos overexpressing MamoAF under the control of maternal *nos-Gal4* driver. Arrowheads indicate Vas mRNA expression in PGCs at stage 14. Scale bar: 10 µm. **b** Embryos were classified into three groups depending on their strong (++), middle (+), and low (-) signal intensities of Vas mRNA in PGCs. **c** Percentages of the embryos carrying PGCs with strong, middle, and low signals in wild type and the embryos overexpressing MamoAF under the control of maternal *nos-Gal4* driver. $^{**}P<0.01$ (Significances are calculated with wild type by Fisher's exact test). **d** Vas mRNA *in situ* hybridisation of wild type and the embryos overexpressing Mamo under the control of maternal *nos-Gal4* driver. Arrowheads indicate Vas mRNA expression in PGCs at stage 15. **e** Embryos were classified into three groups depending on their strong (++), middle (+), and low (-) signal intensities of Vas mRNA in PGCs. **f** Percentages of the embryos carrying PGCs with strong, middle, and low signals in wild type and the embryos overexpressing Mamo under the control of maternal *nos-Gal4* driver. $^{*}P<0.05$ (Significances are calculated with wild type by Fisher's exact test).

a

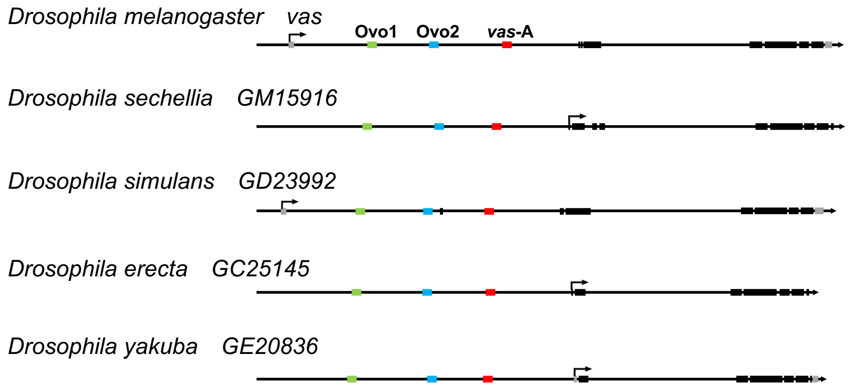

b

	***vas*-A**
Drosophila melanogaster	ATTTGTTTTTCCTGCGTTGCCTGCTGAGT
Drosophila sechellia	TGTTTTTCCTGCGTTGCCTGCTGAGT
Drosophila simulans	TGTTTTTCCTGCGTTGCCTGCTGAGT
Drosophila erecta	TTTTCCTGCGTTGCCCTGCTGAGT
Drosophila yakuba	TGTTTTTCCTGCGTCGCCTGCTGAGT

Supplementary Figure 7 *vas*-A element and Ovo binding consensus sequences in *vas* locus are conserved in several *Drosophila* species**. a** A schematic diagram of *vas* loci in *Drosophila melanogaster*, *Drosophila sechellia*, *Drosophila simulans*, *Drosophila erecta* and *Drosophila yakuba*. **b** *vas*-A element in *Drosophila melanogaster*, *Drosophila sechellia*, *Drosophila simulans*, *Drosophila erecta* and *Drosophila yakuba*. Red indicates Mamo binding consensus sequences.

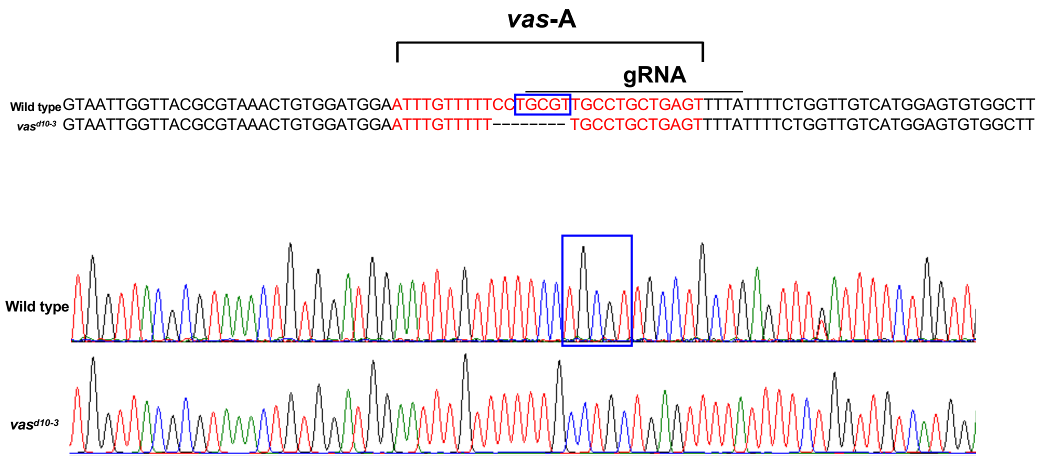

vas-A
gRNA
Wild type GTAATTGGTTACGCGTAAACTGTGGATGGAATTTGTTTTTCCTGCGTTGCCTGCTGAGTTTTATTTTCTGGTTGTCATGGAGTGTGGCTT
vas^{d10-3} GTAATTGGTTACGCGTAAACTGTGGATGGAATTTGTTTTT-------- TGCCTGCTGAGTTTTATTTTCTGGTTGTCATGGAGTGTGGCTT
Wild type
vas^{d10-3}

Supplementary Figure 8 *vas*-A element is deleted in *vas*$^{d10\text{-}3}$ mutant. Sequeneces of the intronic region encompassing *vas*-A element of wild type and *vas*$^{d10\text{-}3}$. Red indicates *vas*-A element. A blue box indicates a Mamo binding consensus sequence. *vas*$^{d10\text{-}3}$ mutation was generated by using CRISPER-Cas9 system. A line indicates the sequence corresponding guide RNA used for the mutagenesis.

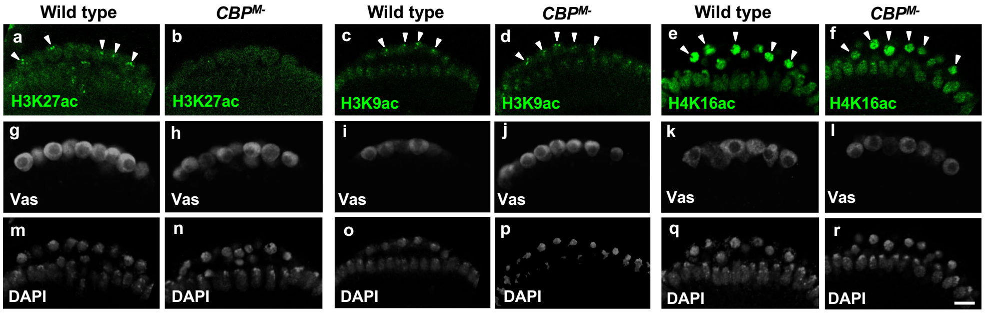

Wild type
CBP^M-
Wild type
CBP^M-
Wild type
CBP^M-
a
H3K27ac
b
H3K27ac
c
H3K9ac
d
H3K9ac
e
H4K16ac
f
H4K16ac
g
Vas
h
Vas
i
Vas
j
Vas
k
Vas
l
Vas
m
DAPI
n
DAPI
o
DAPI
p
DAPI
q
DAPI
r
DAPI

Supplementary Figure 9 Maternal CBP activity is required for H3K27ac accumulation in PGCs. **a**-**r** Wild type embryos and *CBP*$^{M-}$ embryos derived from germline clones homozygous for *nej*$^{0.3}$ mutation at stage 5 are stained for histone modifications (**a**-**f**), Vas (**g**-**l**) and DAPI (**m**-**r**). Embryos are stained for H3K27ac (**a**, **b**), H3K9ac (**c**, **d**) and H4K16ac (**e**, **f**), respectively. Arrowheads indicate signals of the histone modification in PGCs. H3K27ac preferentially marks active enhancers. H3K9ac and H4K16ac, both of which are active histone markers. Scale bar: 10 μm.

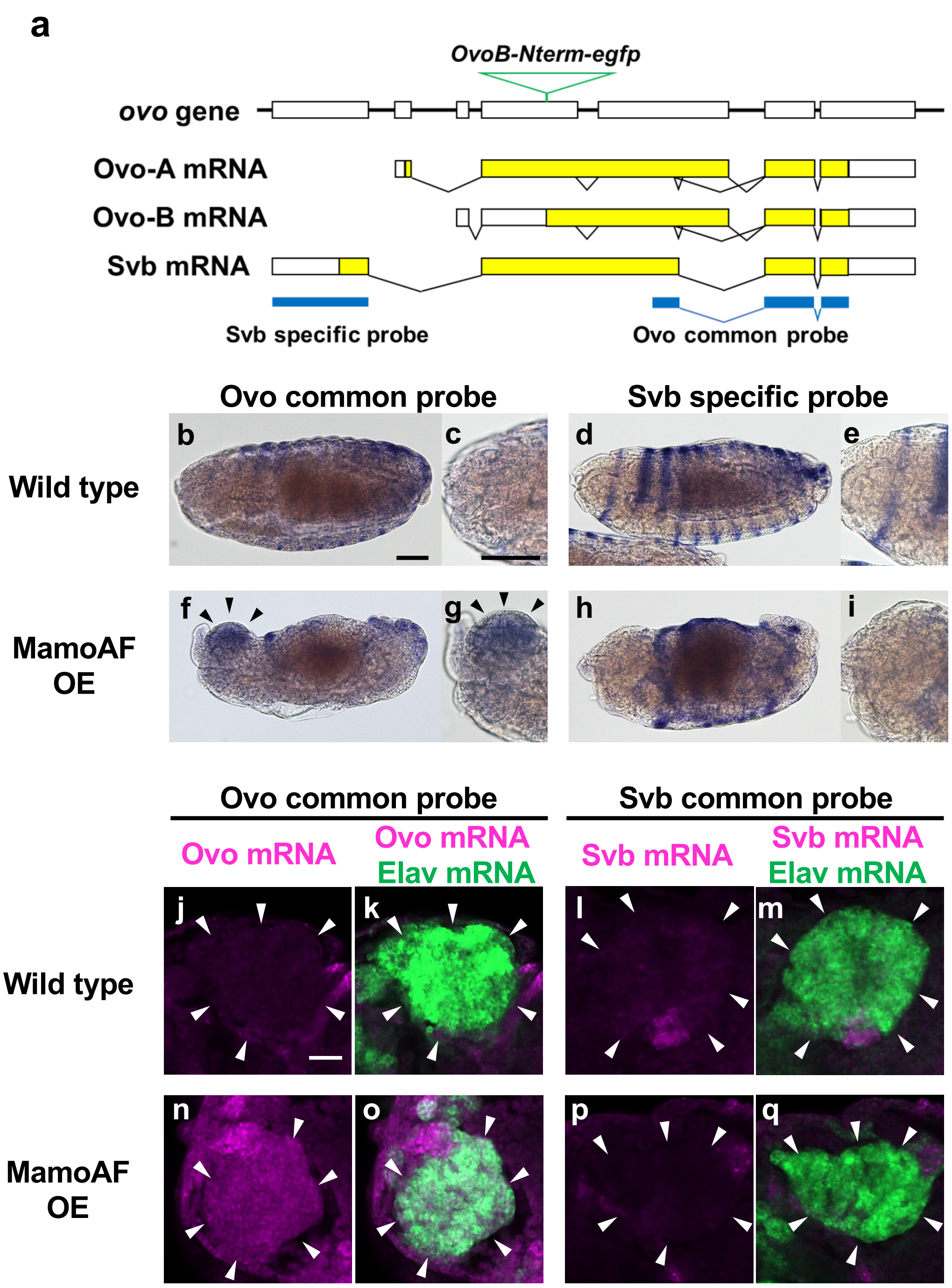
a
OvoB-Nterm-egfp
ovo gene
Ovo-A mRNA
Ovo-B mRNA
Svb mRNA
Svb specific probe
Ovo common probe
Ovo common probe
Svb specific probe
b
c
d
e
Wild type
f
g
h
i
MamoAF OE
Ovo common probe
Svb common probe
Ovo mRNA
Ovo mRNA Elav mRNA
Svb mRNA
Svb mRNA Elav mRNA
j
k
l
m
Wild type
n
o
p
q
MamoAF OE

Supplementary Figure 10 MamoAF induces ectopic Ovo mRNA expression in brain. **a** A schematic diagram of *ovo* locus. **b**-**i** *in situ* hybridisation of wild type embryos and MamoAF OE using an Ovo common probe (**b**, **c**, **f** and **g**) and a Svb specific probe (**d**, **e**, **h** and **i**). Right panels (**c**, **e**, **g** and **i**) are close-up views of brain of left panels (**b**, **d**, **f** and **h**), respectively. Arrowheads indicate ectopic Ovo signals. Scale bar: 50 µm. **j**-**q** Double FISH of wild type embryos and MamoAF OE. Confocal sections of the brain of the embryos hybridised with Ovo and Elav probes (**j**, **k**, **n** and **o**) and with Svb and Elav probes (**l**, **m**, **p** and **q**). Elav is a neural marker. Scale bar: 20 µm.

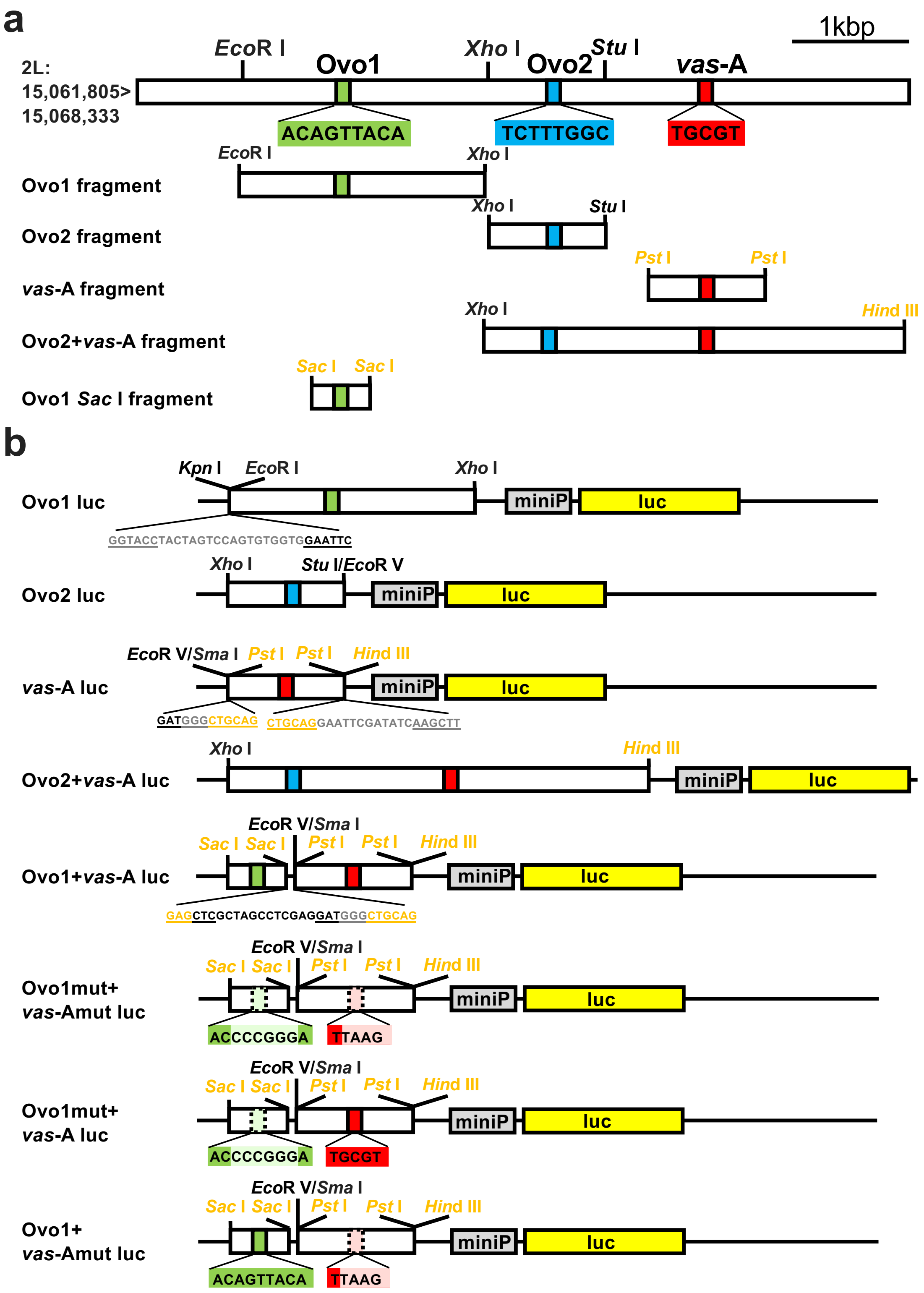
a
1kbp
2L:
15,061,805>
15,068,333
EcoR I
Ovo1
Xho I
Ovo2
Stu I
vas-A
ACAGTTACA
TCTTTGGC
TGCGT
Ovo1 fragment
Ovo2 fragment
vas-A fragment
Ovo2+vas-A fragment
Ovo1 Sac I fragment
Pst I
Hind III
Sac I
b
Ovo1 luc
Kpn I
miniP
luc
GGTACCTACTAGTCCAGTGTGGTGGAATTC
Ovo2 luc
Stu I/EcoR V
vas-A luc
EcoR V/Sma I
GATGGGCTGCAG
CTGCAGGAATTCGATATCAAGCTT
Ovo2+vas-A luc
Ovo1+vas-A luc
GAGCTCGCTAGCCTCGAGGATGGGCTGCAG
Ovo1mut+
vas-Amut luc
ACCCCGGGA
TTAAG
Ovo1mut+
vas-A luc
ACCCCGGGA
TGCGT
Ovo1+
vas-Amut luc
ACAGTTACA
TTAAG

Supplementary Figure 11 The luciferase reporter constructs containing cis-elements in the first intron of *vas*. **a** Schematic representation of the first intron of *vas* (2L: 15,061,805–15,068,333) and the genomic fragments containing the cis-elements used to construct the luciferase reporters. **b** Schematic representation of the luc reporter constructs used in this study. Restriction enzymes and linker sequences are shown. Restriction sites indicated by black are endogenous sites. Yellow indicates the primer-induced restriction sites. Gray box, mini promoter. Yellow box, luciferase reporter gene.

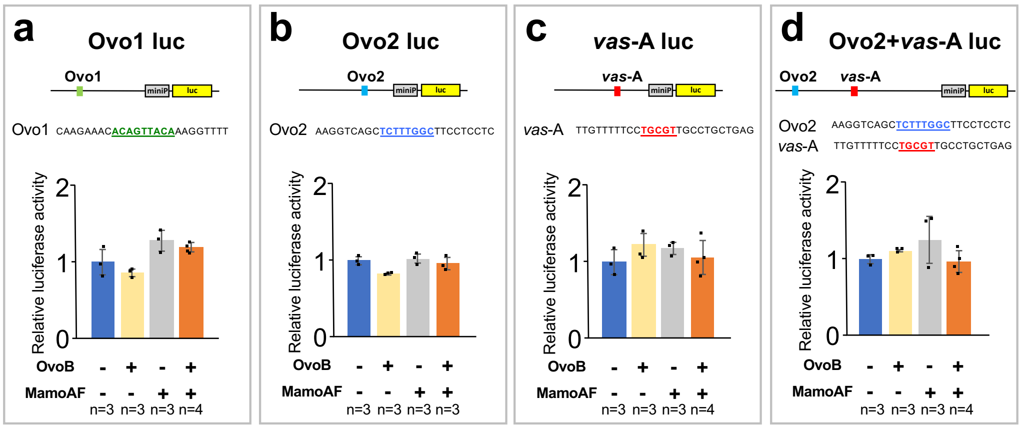

a
Ovo1 luc
Ovo1
miniP
luc
Ovo1 CAAGAAACACAGTTACAAAGGTTTT
Relative luciferase activity
OvoB - + - +
MamoAF - - + +
n=3 n=3 n=3 n=4
b
Ovo2 luc
Ovo2
Ovo2 AAGGTCAGCTCTTTGGCTTCCTCCTC
OvoB - + - +
MamoAF - - + +
n=3 n=3 n=3 n=3
c
vas-A luc
vas-A
vas-A TTGTTTTTCCTGCGTTGCCTGCTGAG
OvoB - + - +
MamoAF - - + +
n=3 n=3 n=3 n=4
d
Ovo2+vas-A luc
Ovo2 vas-A
Ovo2 AAGGTCAGCTCTTTGGCTTCCTCCTC
vas-A TTGTTTTTCCTGCGTTGCCTGCTGAG
OvoB - + - +
MamoAF - - + +
n=3 n=3 n=3 n=4

Supplementary Figure 12 The individual cis-elements in the first intron of *vas* does not response to MamoAF and OvoB. The luciferase reporter constructs containing the *vas* intronic fragments, Ovo1 luc, Ovo2 luc, *vas*-A luc and Ovo2+*vas*-A luc were used for reporter assays. The mean of relative luciferase activities and SD are shown.

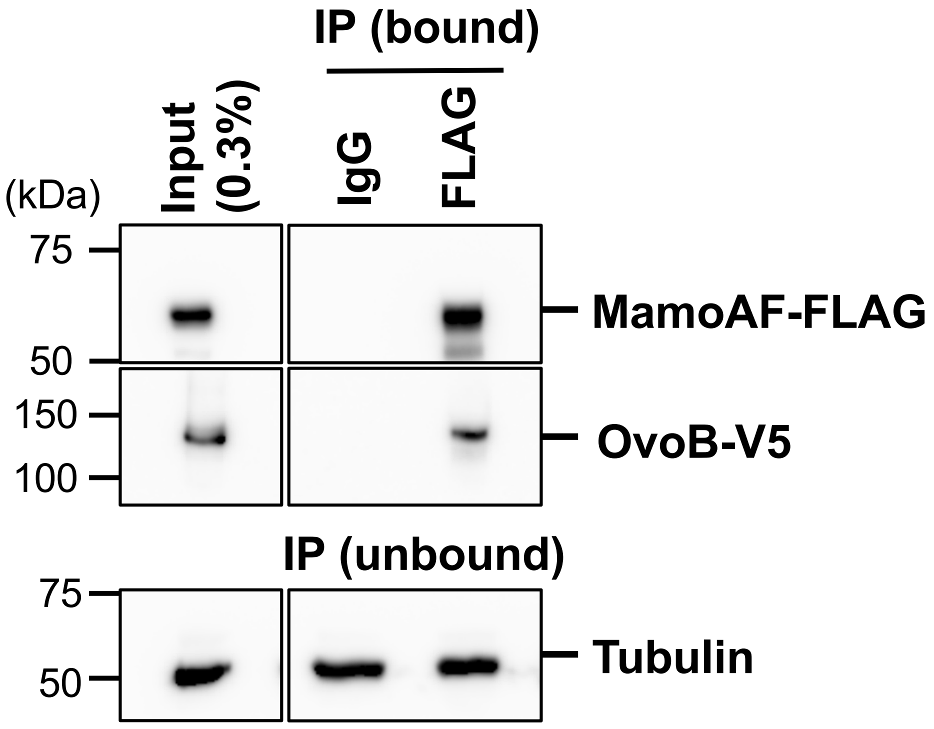
IP (bound)
Input (0.3%)
IgG
FLAG
(kDa)
75
50
150
100
MamoAF-FLAG
OvoB-V5
IP (unbound)
75
50
Tubulin

Supplementary Figure 13 MamoAF interacts with OvoB. Immunoprecipitation assays with nuclear extracts of S2 cells that had been transfected with both FLAG-tagged MamoAF and V5-tagged OvoB. The precipitate with a FLAG antibody were analysed by FLAG and V5 antibodies.

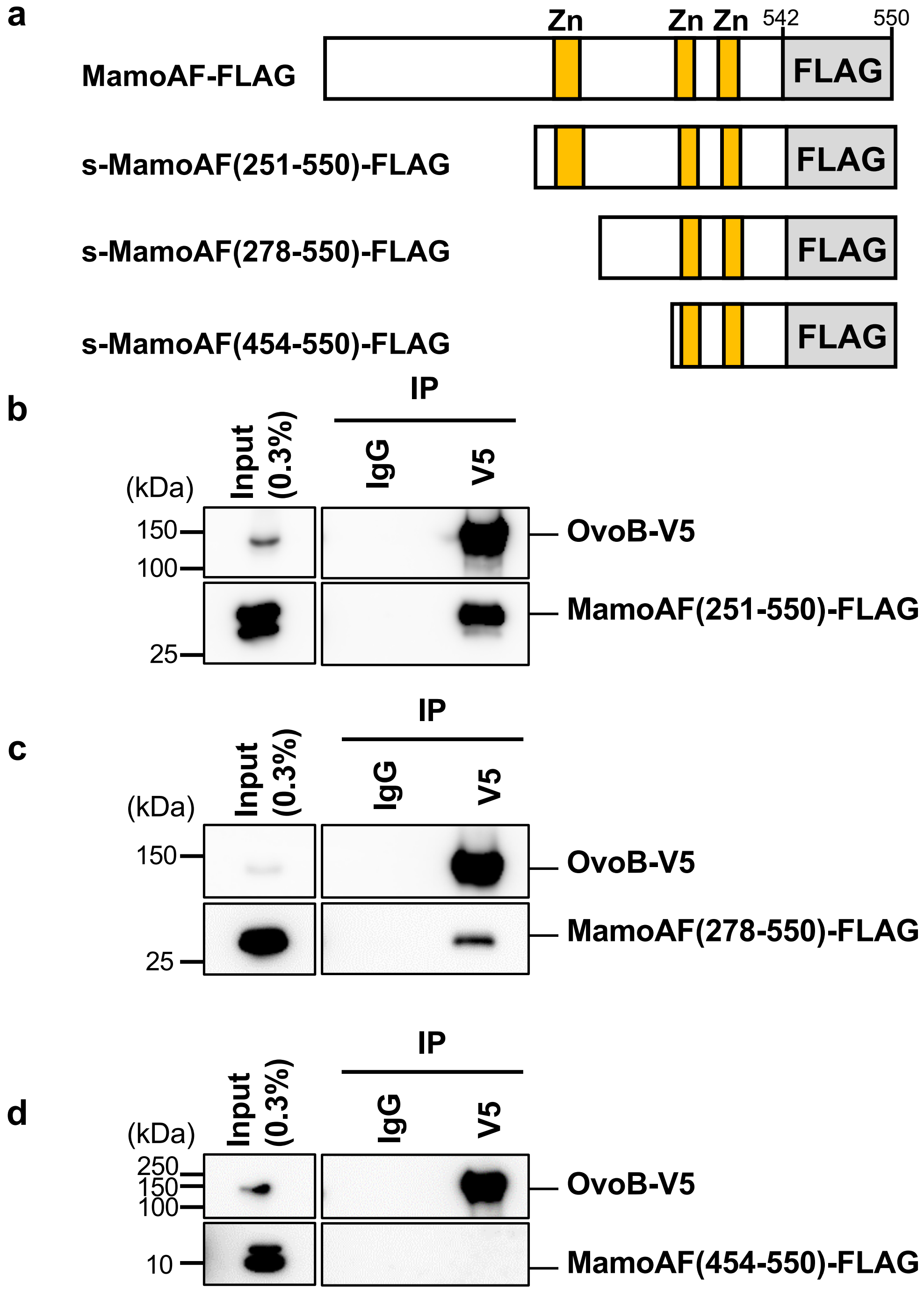

a
Zn
Zn
Zn
542
550
MamoAF-FLAG
FLAG
s-MamoAF(251-550)-FLAG
FLAG
s-MamoAF(278-550)-FLAG
FLAG
s-MamoAF(454-550)-FLAG
FLAG
b
IP
Input (0.3%)
IgG
V5
(kDa)
150
100
25
OvoB-V5
MamoAF(251-550)-FLAG
c
IP
Input (0.3%)
IgG
V5
(kDa)
150
25
OvoB-V5
MamoAF(278-550)-FLAG
d
IP
Input (0.3%)
IgG
V5
(kDa)
250
150
100
10
OvoB-V5
MamoAF(454-550)-FLAG

Supplementary Figure 14 N-terminal region of MamoAF is required for the interaction with OvoB. **a** A schematic diagram of FLAG-tagged MamoAF fragments used for immunoprecipitation assays. **b**-**d** Immunoprecipitation assays with nuclear extracts of S2 cells that had been transfected with both V5-tagged OvoB and FLAG-tagged MamoAF fragments: MamoAF(251-550)-FLAG (**b**), MamoAF(278-550)-FLAG (**c**) and MamoAF(454-550)-FLAG (**d**).

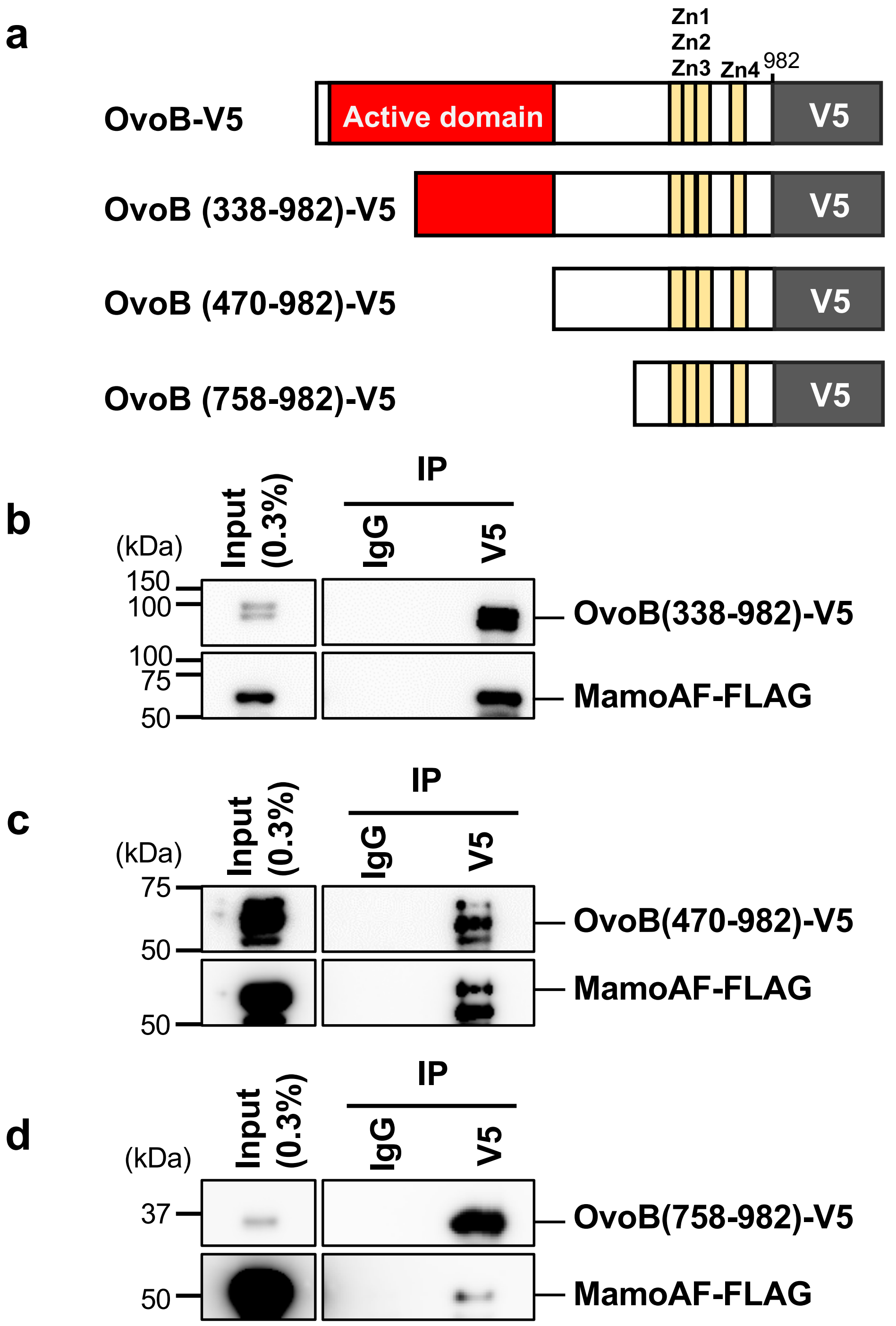

a
b
c
d
OvoB-V5
OvoB (338-982)-V5
OvoB (470-982)-V5
OvoB (758-982)-V5
Active domain
Zn1
Zn2
Zn3
Zn4
982
V5
Input (0.3%)
IP
IgG
V5
(kDa)
150
100
75
50
37
OvoB(338-982)-V5
MamoAF-FLAG
OvoB(470-982)-V5
OvoB(758-982)-V5

Supplementary Figure 15 N-terminal region of OvoB enhances the interaction between MamoAF and OvoB. **a** A schematic diagram of V5-tagged OvoB fragments used for immunoprecipitation assays. **b-d** Immunoprecipitation assays with nuclear extracts of S2 cells that had been transfected with both MamoAF-FLAG and V5-tagged OvoB fragments: OvoB(338-982)-V5 (**b**), OvoB(470-982)-V5 (**c**) and OvoB(758-982)-V5 (**d**).

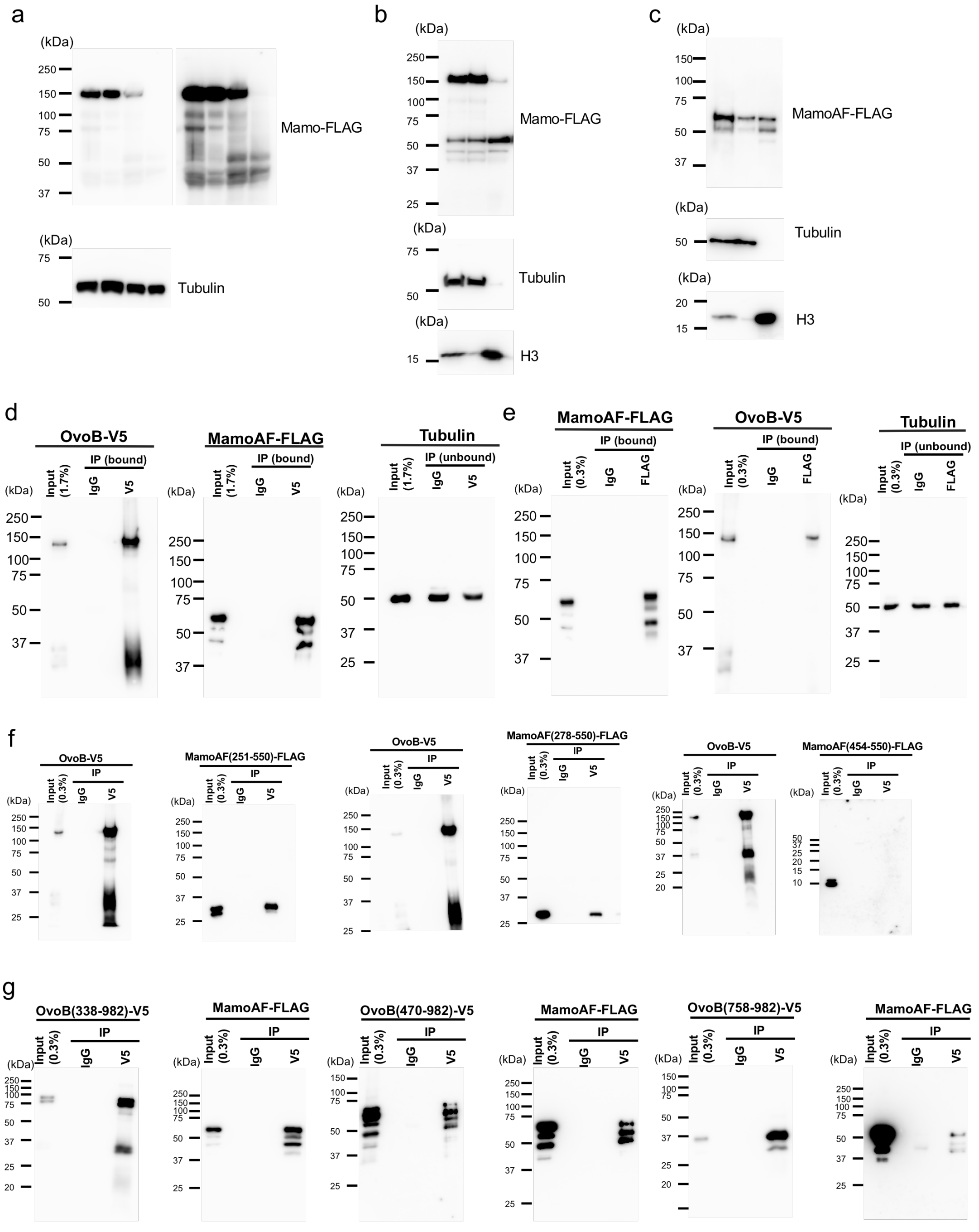
a
(kDa)
250
150
100
75
50
37
Mamo-FLAG
(kDa)
75
50
Tubulin
b
(kDa)
250
150
100
75
50
37
25
Mamo-FLAG
(kDa)
75
50
Tubulin
(kDa)
15
H3
c
(kDa)
150
100
75
50
37
MamoAF-FLAG
(kDa)
50
Tubulin
(kDa)
20
15
H3
d
OvoB-V5
MamoAF-FLAG
Tubulin
IP (bound)
IP (unbound)
Input (1.7%)
IgG
V5
(kDa)
250
150
100
75
50
37
25
e
MamoAF-FLAG
OvoB-V5
Tubulin
IP (bound)
IP (unbound)
Input (0.3%)
IgG
FLAG
f
OvoB-V5
MamoAF(251-550)-FLAG
MamoAF(278-550)-FLAG
MamoAF(454-550)-FLAG
IP
Input (0.3%)
IgG
V5
g
OvoB(338-982)-V5
MamoAF-FLAG
OvoB(470-982)-V5
OvoB(758-982)-V5
IP
Input (0.3%)
IgG
V5

Supplementary Figure 16 Original blots.
a Original blots of Figure 1a. Membranes were cut before incubation with the antibody.
b Original blots of Figure 1b. Membranes were cut before incubation with the antibody.
c Original blots of Figure 1c. Membranes were cut before incubation with the antibody.
d Original blots of Figure 6g.
e Original blots of Supplementary Figure 13.
f Original blots of Supplementary Figure 14.
g Original blots of Supplementary Figure 15.

Supplementary Table 1

Differentiation of egg chambers containing *mamo* germline clones can be rescued by overexpressing full-length Mamo-FLAG and MamoAF-FLAG

Genotype[a]	Percentages of ovarioles with differentiating egg chambers (n) [e]
Wild type	97.7 (128)
mamo^{SVA53}/*mamo*^{SVA53}, *nos-Gal4*[b]	1.4 (74)
mamo^{SVA53}/*mamo*^{SVA53}, *UASp-Mamo-FLAG, nos-Gal4*[c]	28.8 (73)
mamo^{SVA53}/*mamo*^{SVA53}, *UASp-MamoAF-FLAG, nos-Gal4*[d]	13.2 (197)

[a] Genotypes of females.
[b] Females with germline clones homozygous for the *mamo* mutation and carrying *nos-Gal4* driver were dissected and the ovaries were examined.
[c] Females with germline clones homozygous for the *mamo* mutation and carrying both *UASp-Mamo-FLAG* and *nos-Gal4* driver were dissected and the ovaries were examined.
[d] Females with germline clones homozygous for the *mamo* mutation and carrying both *UASp-MamoAF-FLAG* and *nos-Gal4* driver were dissected and the ovaries were examined.
[e] Ovarioles containing egg chambers after oogenic stage 6 were scored.

Supplementary Table 2

Differentiation of mature eggs derived from *mamo* germline clones can be rescued by overexpressing full-length Mamo-FLAG but not MamoAF-FLAG

Maternal genotype[a]	Eggs/h/female[e]	n	Hatching (%)[f]	n
Wild type	1.07 ± 0.37	8	92.1 ± 1.5	8
mamo^{SVA53}/*mamo*^{SVA53}, *nos-Gal4*[b]	0	4	-	-
mamo^{SVA53}/*mamo*^{SVA53}, *UASp-Mamo-FLAG, nos-Gal4*[c]	0.29 ± 0.1	4	36.9 ± 9.1	4
mamo^{SVA53}/*mamo*^{SVA53}, *UASp-MamoAF-FLAG, nos-Gal4*[d]	0	4	-	-

[a] Genotypes of females.
[b] Females with germline clones homozygous for the *mamo* mutation and carrying *nos-Gal4* driver were used.
[c] Females with germline clones homozygous for the *mamo* mutation and carrying both *UASp-Mamo-FLAG* and *nos-Gal4* driver were used.
[d] Females with germline clones homozygous for the *mamo* mutation and carrying both *UASp-MamoAF-FLAG* and *nos-Gal4* driver were used.
[e] Females with each genotype were mated with *y w* males. 30–77 females were used. Eggs produced by the females were scored. Standard deviation is given.
[f] 30–151 embryos were collected, and incubated at 25 °C for 1 day. Hatching rate of embryos was scored. Standard deviation is given.
