# Supplementary material for: A truncated form of a transcription factor Mamo activates vasa in Drosophila embryos
Source: Commun Biol. 2019 Nov 20;2:422. doi: 10.1038/s42003-019-0663-4 (PMC6868150; doi:10.1038/s42003-019-0663-4)
Supplement: Supplementary file 2 — Description of Additional Supplementary Files [file 42003_2019_663_MOESM2_ESM.docx]

Description of additional supplementary items

SUPPLEMENTARY DATA FILE LEGEND

Supplementary Data 1. Data for figures used in this study. Each tab corresponds to a given Figures.
