# Supplementary material for: A truncated form of a transcription factor Mamo activates vasa in Drosophila embryos
Source: Commun Biol. 2019 Nov 20;2:422. doi: 10.1038/s42003-019-0663-4 (PMC6868150; doi:10.1038/s42003-019-0663-4)
Supplement: Supplementary file 4 — Reporting Summary [file 42003_2019_663_MOESM4_ESM.pdf]

nature research

Corresponding author(s): Masanori Mukai

Last updated by author(s): Oct 5, 2019

Reporting Summary

Nature Research wishes to improve the reproducibility of the work that we publish. This form provides structure for consistency and transparency in reporting. For further information on Nature Research policies, see Authors & Referees and the Editorial Policy Checklist.

Statistics

For all statistical analyses, confirm that the following items are present in the figure legend, table legend, main text, or Methods section.

n/a	Confirmed	
☐	☒	The exact sample size (*n*) for each experimental group/condition, given as a discrete number and unit of measurement
☐	☒	A statement on whether measurements were taken from distinct samples or whether the same sample was measured repeatedly
☐	☒	The statistical test(s) used AND whether they are one- or two-sided<br>*Only common tests should be described solely by name; describe more complex techniques in the Methods section.*
☒	☐	A description of all covariates tested
☒	☐	A description of any assumptions or corrections, such as tests of normality and adjustment for multiple comparisons
☐	☒	A full description of the statistical parameters including central tendency (e.g. means) or other basic estimates (e.g. regression coefficient) AND variation (e.g. standard deviation) or associated estimates of uncertainty (e.g. confidence intervals)
☐	☒	For null hypothesis testing, the test statistic (e.g. *F*, *t*, *r*) with confidence intervals, effect sizes, degrees of freedom and *P* value noted<br>*Give P values as exact values whenever suitable.*
☒	☐	For Bayesian analysis, information on the choice of priors and Markov chain Monte Carlo settings
☒	☐	For hierarchical and complex designs, identification of the appropriate level for tests and full reporting of outcomes
☒	☐	Estimates of effect sizes (e.g. Cohen's *d*, Pearson's *r*), indicating how they were calculated

Our web collection on statistics for biologists contains articles on many of the points above.

Software and code

Policy information about availability of computer code

Data collection	Fluorescent imaging was acquired with Zen 2.1 Software (Zeiss) or the software for the FV1200(Olympus).<br>Western blotting imaging was acquired with the software for the ImageQuant LAS 4000 system (GE healthcare).
Data analysis	Expression levels of vasa were quantified using the software for Thermal Cycler Dice TP700 (Takara).<br>Microsoft Excel was used for the statistical analyses.

For manuscripts utilizing custom algorithms or software that are central to the research but not yet described in published literature, software must be made available to editors/reviewers. We strongly encourage code deposition in a community repository (e.g. GitHub). See the Nature Research guidelines for submitting code & software for further information.

Data

Policy information about availability of data

All manuscripts must include a data availability statement. This statement should provide the following information, where applicable:

- Accession codes, unique identifiers, or web links for publicly available datasets
- A list of figures that have associated raw data
- A description of any restrictions on data availability

Fig. 1a Raw data files (5 files)
Fig. 1b Raw data file (6 file)
Fig. 1c Raw data file (6 file)
Fig. 1d Raw data files (1 file)
Fig. 1 e Raw data files (11 files)
Fig. 1 f Raw data files (15 files)

Fig. 2b Raw data files (2 files)
Fig. 2c Raw data files (1 file)
Fig. 2d Raw data files (3 files)
Fig. 2e Raw data file (1 file)
Fig. 2f Raw data file (1 file)
Fig. 2g Raw data files (2 files)
Fig. 2h Raw data files (1 file)

Fig. 3a Raw data files (8 files)

Fig. 3b Raw data files (32 files)

Fig. 4 Raw data files (12 files)

Fig. 5a, b Raw data files (4 files)
Fig. 5c, d Raw data files (3 files)

Fig. 6 b c d e Raw data files (1 file)
Fig. 6 f Raw data files (1 file)
Fig. 6 g Raw data files (6 files)

sFig. 1 Raw data file (1 file)
sFig. 2 Raw data file (13 files)
sFig. 3 Raw data file (9 files)
sFig. 4 Raw data file (18 files)

sFig. 5 Raw data files (32 files)
sFig. 6 a-c Raw data files (6 files)
sFig. 6 d-f Raw data files (6 files)

sFig. 9 Raw data files (18 file)
sFig. 10 b-i Raw data files (8 files)
sFig. 10 j-q Raw data files (8 files)
sFig. 13 Raw data files (1 file)

sFig. 14 Raw data files (6 files)
sFig. 15 Raw data files (12 files)
sFig. 16 Raw data files (12 files)

All raw data are available if they are requested.

Field-specific reporting

Please select the one below that is the best fit for your research. If you are not sure, read the appropriate sections before making your selection.

☒ Life sciences ☐ Behavioural & social sciences ☐ Ecological, evolutionary & environmental sciences

For a reference copy of the document with all sections, see nature.com/documents/nr-reporting-summary-flat.pdf

Life sciences study design

All studies must disclose on these points even when the disclosure is negative.

Sample size	Sample size for each experiment is indicated in the figure legends. The sample size was chosen based on previous experience for each experiment to yield high power to detect specific effects.<br>No statistical methods were used to predetermine sample size.
Data exclusions	Basically, no samples were excluded from experiments. But, the embryos that obviously show abnormal embryonic development and have not progressed to a specific stage (embryonic stage 5) are not used for analysis. Contaminated S2 cell cultures were excluded from experiments (sFig 12-15 and Fig. 6 b-g).
Replication	Replicate experiments were successful.
Randomization	No randomization of flies.<br>Flies were chosen based on morphological markers to determine the genotypes. The embryos derived from the female flies with different

genetic back ground (ex. wild type and MamoAF overexpressed embryos) were collected, fixed and stained at the same time to decrease technical and environmental variance.

Blinding

Investigators were not blinded to fly genotypes during experiments.
Each sample group with different genotypes was labeled but treated at random during the staining and observation.

Reporting for specific materials, systems and methods

We require information from authors about some types of materials, experimental systems and methods used in many studies. Here, indicate whether each material, system or method listed is relevant to your study. If you are not sure if a list item applies to your research, read the appropriate section before selecting a response.

Materials & experimental systems

n/a	Involved in the study
☐	☒ Antibodies
☐	☒ Eukaryotic cell lines
☒	☐ Palaeontology
☒	☐ Animals and other organisms
☒	☐ Human research participants
☒	☐ Clinical data

Methods

n/a	Involved in the study
☒	☐ ChIP-seq
☒	☐ Flow cytometry
☒	☐ MRI-based neuroimaging

Antibodies

Antibodies used

For immunostaining
rabbit anti-Vas antibody (Dr. S. Kobayashi)
mouse anti-Elav monoclonal antibody (Developmental Studies Hybridoma Bank)
mouse anti-GFP monoclonal antibody (3E6, Thermo Fisher)
mouse anti-H3K27ac monoclonal antibody (Dr. H. Kimura).
mouse anti-H3K9ac monoclonal antibody (Dr. H. Kimura).
mouse anti-H4K16ac monoclonal antibody (Dr. H. Kimura).
mouse anti-Piwi monoclonal antibody (Dr. M. Shiomi).
mouse anti-FLAG M2 antibody (Sigma, F1804).

AlexaFlour-conjugated highly cross-absorbed antibodies (Molecular Probes)
(A11036, A11001, A11077, A11034, A11031)

For WB
mouse anti-FLAG M2 antibody (Sigma, F1804)
mouse anti-V5 antibody (abcam, ab27671)
mouse anti-tubulin antibody (Sigma, T6199)
rabbit anti-histone H3 (abcam, ab1791)

horseradish peroxidase (HRP)-conjugated mouse IgG antibody (Bio-Rad, immune-star anti-mouse HRP)
horseradish peroxidase (HRP)-conjugated rabbit IgG antibody (Bio-Rad, immune-star anti-rabbit HRP)

for FISH
anti-DIG alkaline phosphatase-conjugated antibody (Roche)
anti-FITC horseradish peroxidase-conjugate antibody (Perkin Elmer)

for IP
rabbit anti-V5 antibody (Abcam, ab9116)
rabbit anti-FLAG antibody (Sigma, F7425)
rabbit control IgG (Cell signaling technology, 2729S)

Validation

The antibodies used in this study are used in previous studies, and the staining pattern has been analyzed. Judging from the staining pattern, it was judged that all the antibodies properly recognized the antigen.

Eukaryotic cell lines

Policy information about cell lines

Cell line source(s)	Drosophila S2 cells were kindly gifted from Dr. Kageyama of Kobe University.
Authentication	We used single S2 cell line in biochemical studies. The S2 cells were not authenticated.
Mycoplasma contamination	The S2 cells were not tested for mycoplasma contamination.
Commonly misidentified lines (See ICLAC register)	n/a
